# Controlling surface morphology and sensitivity of granular and porous silver films for surface-enhanced Raman scattering, SERS

**DOI:** 10.3762/bjnano.9.263

**Published:** 2018-11-07

**Authors:** Sherif Okeil, Jörg J Schneider

**Affiliations:** 1 Eduard-Zintl-Institut für Anorganische und Physikalische Chemie, Technische Universität Darmstadt, Alarich-Weiss-Straße 12, 64287 Darmstadt, Germany

**Keywords:** plasma treatment, silver, sputtering, surface-enhanced Raman spectroscopy (SERS), surface roughening

## Abstract

The design of efficient substrates for surface-enhanced Raman spectroscopy (SERS) for large-scale fabrication at low cost is an important issue in further enhancing the use of SERS for routine chemical analysis. Here, we systematically investigate the effect of different radio frequency (rf) plasmas (argon, hydrogen, nitrogen, air and oxygen plasma) as well as combinations of these plasmas on the surface morphology of thin silver films. It was found that different surface structures and different degrees of surface roughness could be obtained by a systematic variation of the plasma type and condition as well as plasma power and treatment time. The differently roughened silver surfaces act as efficient SERS substrates showing greater enhancement factors compared to as prepared, sputtered, but untreated silver films when using rhodamine B as Raman probe molecule. The obtained roughened silver films were fully characterized by scanning electron microscopy (SEM), atomic force microscopy (AFM), X-ray diffraction (XRD), transmission electron microscopy (TEM), X-ray photoelectron (XPS and Auger) and ultraviolet–visible spectroscopy (UV–vis) as well as contact angle measurements. It was found that different morphologies of the roughened Ag films could be obtained under controlled conditions. These silver films show a broad range of tunable SERS enhancement factors ranging from 1.93 × 10^2^ to 2.35 × 10^5^ using rhodamine B as probe molecule. The main factors that control the enhancement are the plasma gas used and the plasma conditions, i.e., pressure, power and treatment time. Altogether this work shows for the first time the effectiveness of a plasma treatment for surface roughening of silver thin films and its profound influence on the interface-controlled SERS enhancement effect. The method can be used for low-cost, large-scale production of SERS substrates.

## Introduction

The great enhancement of Raman signals obtained from molecules when they are in close vicinity to a rough noble-metal surface (e.g., gold, silver and copper) has attracted a great deal of interest in the last decades [1]. This phenomenon, called surface-enhanced Raman scattering [2–3], depends on the fact that incident light leads to the excitation of surface plasmon resonances, which in turn lead to a concentration of the incident electromagnetic field thus enhancing the Raman scattering effect. This effect is even further enhanced by the presence of so called hot spots, which are sub-10 nm gaps where the electromagnetic field is further magnified due to constructive interference of the plasmon resonances [4]. Electromagnetic enhancement is the main reason for the observed Raman enhancement and depends on the local electromagnetic field at the metal surface while the chemical enhancement depends on the analyte itself and results from an effective charge transfer between the noble metal and the adsorbed probe molecule [5–6].

While the Raman effect is intrinsically weak and typically does not permit the analysis of low concentrations, the SERS signal dramatically enhances the sensitivity typically by orders of magnitude and allows for the analysis of low analyte concentrations [5,7]. The fabrication of SERS substrates began with electrochemical oxidation/reduction cycles especially of silver electrodes. This is the most widely studied synthesis and is still subject to further investigation and optimization to obtain more efficient SERS substrates [2,8–11]. One modification of the electrochemical fabrication of SERS substrates involves the electrochemical etching of silver to obtain porous silver nanostructures [12]. Other routes include the use of gold or silver nanoparticles of different shapes in solution and their assembly on a solid substrate [6,13–17], nanosphere lithography [18–25] as well as nanolithography and nanoimprinting [26–34]. Additional methods appeared in which nanoparticles or metal films are deposited on structured substrates as carbon nanotubes [35–39], graphene foam [40], nanorod or nanopillar arrays [41–42], biological scaffolds [43–44], black silicon [45–46], anisotropically etched single-crystal silicon [47], plasma-treated plastic [48] and anodic aluminum oxide films [49–52]. Some methods aim at the fabrication of three -dimensional silver or gold structures, such as oblique-angle vapor deposition used for the fabrication of silver nanorod arrays [53–56], and nanotransfer printing, which was used to build stacks of gold nanorods or nanowires [4]. The production of nanoporous metal films or particles through a dealloying process also emerged as an effective tool for the facile formation of a large number of SERS-active hot spots [57–60]. Recently, other methods emerged with the aim of producing SERS substrates at low cost, enabling their large-scale production. These methods include inkjet-printing and pen-on-paper approaches [61–62].

Plasma treatment has been widely used for the last decades for microelectronics and surface modification in industry [63–64]. A large variety of plasmas exist depending on the excitation source, the operating pressure and the device geometry [63,65]. Advantages of the use of a radio frequency (rf) plasma for chemical modification is that no hazardous chemicals and solvents are involved. Moreover, it enables a quick chemical modification under ambient conditions in the gas phase [64,66–71]. Thus, plasma treatment could present a straightforward and affordable alternative to the electrochemical roughening of silver and can be even used as a technological reliable alternative for the production of SERS substrates. Interestingly, up to date the use of plasma treatment for SERS applications is still limited [72–73] despite its high potential in modifying metal surfaces. Especially a systematic study is lacking.

Herein we report on our studies towards a systematic investigation of the effect of different rf plasmas on sputtered silver surfaces to determine the potential of plasma treatment in a controlled increase of the surface roughness of silver as well as the formation of hot spots on the silver surface for the use in SERS. To the best of our knowledge this is the first systematic investigation in that direction.

## Experimental

### Preparation of sputtered silver films

Thin films of silver were sputtered on glass substrates cleaned with acetone. Different thicknesses of silver were sputtered using a benchtop Cressington 208HR sputter coater in which the thickness of the sputtered silver layer was monitored by a MTM-20 high-resolution thickness controller. The sputtered silver films are named according to the thickness indicated by the thickness controller. A silver target (99.99%) from Evochem with 57 mm diameter and 0.2 mm thickness was used as sputter target. The deposition rate used was about 0.4 nm/s at a chamber pressure of about 0.05 mbar.

### Plasma treatment of the prepared silver films

The sputtered silver films of different thicknesses were treated with different plasma gases using a capacitively coupled rf plasma apparatus operated at 13.56 MHz (Diener electronic, model Femto, Germany) with a maximum power of 200 W. After introduction of the sample into the plasma chamber, where the sample was placed on the lower electrode, the chamber was evacuated to a pressure below 0.2 mbar. Then the gas to be used for plasma treatment was introduced at a specified flow rate, controlled by a mass flow controller. When the pressure in the plasma chamber achieved its equilibrium the radio frequency controller was switched on at a specified power and left for a defined period of time to obtain the desired plasma treatment for the silver films. The operating temperature was set at 25 °C but heating occurred during the plasma treatment depending on the plasma treatment time. The gases used for plasma treatment were argon at a pressure of 1.5 mbar for a flow rate of 16.7 sccm, nitrogen at a pressure of 0.8 mbar for a flow rate of 12 sccm, hydrogen at a pressure of 0.22 mbar for a flow rate of 6 sccm and 0.38 mbar for a flow rate of 12 sccm, oxygen at a pressure of 0.4 mbar for a flow rate of 6 sccm and 0.8 mbar for a flow rate of 12 sccm, and finally air at a pressure of 0.8 mbar for a flow rate of 12 sccm. When describing the experiments, the used gases get a notation according to the gas flow and power used for the plasma treatment, i.e., a plasma gas with the notation g12-p200 indicates a gas flow of 12 sccm and a power of 200 W, which was used for almost all samples unless otherwise indicated. For simplicity, any sample without notation for gas flow and power has been synthesized using 12 sccm gas flow and a power of 200 W except for argon plasma where the standard gas flow used is 16.7 sccm. Samples obtained under other conditions will get a notation stating the gas flow and power used.

### Characterization of the as-prepared silver films

The prepared silver films were characterized using atomic force microscopy (AFM) in contact mode on a CP-II AFM (Bruker-Veeco) with SiC cantilevers to determine the topography and surface roughness (root mean square roughness, *R*_q_). Scanning electron microscopy (SEM) of the silver films was performed on a Philips XL-30 FEG using an electron beam at 25 kV. Cross-sectional transmission electron microscopy (TEM) images of the silver films were recorded using a Tecnai G2 F20 microscope operating at 200 kV after the use of focused-ion beam (FIB) for sample preparation [74]. X-ray diffraction (XRD) was performed on a Rigaku Miniflex 600@40 kV 15 mA diffractometer using Cu Kα_1_ radiation (λ = 1.541 Å). XPS measurements were performed using a K-Alpha XPS spectrometer (ThermoFischer Scientific, East Grinstead, UK). Data acquisition and processing was done using the Thermo Avantage software. All samples were analyzed using a microfocused, monochromated Al Kα X-ray source (30–400 µm spot size). The K-Alpha charge compensation system was employed during analysis, using electrons of 8 eV energy and low-energy argon ions to prevent any localized charge build-up. Auger spectroscopy was performed using a PHI 680 (Physical Electronics) scanning Auger nanoprobe operated at an acceleration voltage of 20 keV and a current of 10 nA. Sputtering was carried out under ultra-high vacuum (5 × 10^−9^ Torr), with an argon gun operated at 250 eV and 500 nA. The UV–vis spectra of the silver films on glass substrate were recorded by a Thermo Scientific UV–vis spectrophotometer (Evolution 600). The water contact angle was measured using a Krüss DSA 30 model drop-shape analysis system. The water contact angle was measured by placing 5 µL water droplets on the silver surface.

### Atomic layer deposition (ALD) of Al_2_O_3_

Silver films on glass substrates were inserted into the ALD chamber (Savannah G2, Ultratech/CNT, Cambridge Nanotech) for deposition of a 1 nm thick layer of Al_2_O_3_ using trimethylaluminium (98+%, Strem Chemicals, Inc.) and water (HPLC grade, Sigma Aldrich) as precursors at 200 °C. The precursors were vaporized at room temperature and 20 sccm argon was used as carrier gas. The exposure time for both, trimethylaluminium and water, were 0.015 s and the flushing between the precursor pulses was done with argon for 5 s. For deposition of 1 nm Al_2_O_3_ ten cycles were carried out.

### SERS measurements on the prepared silver films

Rhodamine B (RhB) was used as Raman probe to compare the performance of the different silver films. A 10^−6^ M solution of RhB was prepared in deionized water. A sufficient amount of this solution was dropped on the surface of the silver SERS substrates to cover the surface completely. The SERS substrate was left for 30 min in the aqueous RhB solution to enable the adsorption of the RhB on the silver surface. After that the SERS substrate was rinsed with deionized water to remove any excess of the RhB. The performance of the different SERS substrates was then evaluated through the measurement of the SERS spectra of RhB using a Horiba Jobin-Yvon LabRAM HR800 micro-Raman spectrometer employing a 514.5 nm Ar laser and a 10× objective (NA = 0.25), and a measurement time of 30 s with a laser power of 1.0 mW. Alternatively, a 632.8 nm HeNe laser using a 50× LWD objective (NA = 0.5) and a measurement time of 30 s with a laser power of 2.0 mW was used. For Raman mapping as well as single scans another Raman setup (Alpha300 R micro-Raman, WITec, Germany) with a Nd:YAG 532 nm laser was used. The samples were exposed to the laser with a power of 1.0 mW through a 50× microscope objective (NA = 0.80) resulting in a spot size of about 0.8 µm. SERS spectra were collected using 1 s as integration time for single scans. For Raman mapping a 30 µm × 30 µm area was scanned, collecting 900 points (30 lines with 30 points each). The same parameters as for single scans were used. A commercial SERS substrate provided from Abacus (Analytical Systems GmbH) consisting of silver nanoparticles on a paper substrate was used for comparison.

## Results and Discussion

### Processing and characterization of sputtered and plasma-treated silver films

Radio frequency plasma is generated through exposing the feed gas to an external radio frequency field using a frequency of 13.56 MHz. The setup consists simply of two parallel plates about 10 cm apart where the substrate is placed on the bottom electrode. These electrodes are connected to radio frequency generator generating the alternating electric field at 13.56 MHz frequency (Scheme S1, Supporting Information File 1). At this frequency, electrons quickly respond to any minor changes in the electric field thus gaining a significant amount of energy. When these highly energetic electrons collide with the feed gas atoms or molecules this results into a series of successive complex processes, namely ionization (1 and 2), excitation (3) and relaxation (4) as well as dissociation in case of diatomic species (5) [75]:


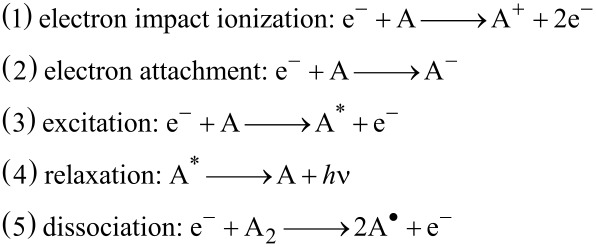


**Scheme 1:**
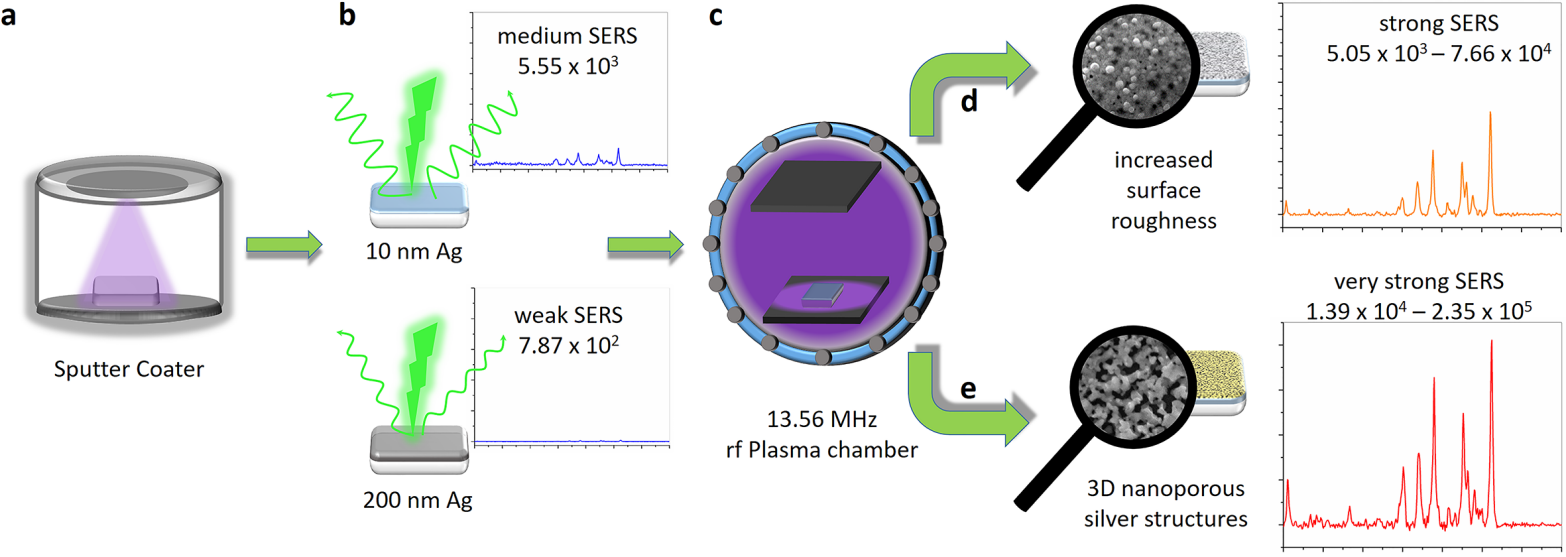
Overview of the steps involved in the fabrication of the SERS substrates using different rf plasma treatments. (a) 25 s of sputtering leads to a transparent silver film of 10 nm thickness. 8 min of sputtering leads to a non-transparent silver film of 200 nm thickness. (b) The resulting silver films have different SERS activities; the ultrathin film has a higher SERS activity than the thicker silver film. (c) Plasma treatment of the sputtered silver films in a 13.56 MHz rf plasma chamber with different plasma gases in order to increase the surface roughness and nanoporosity of the silver films. (d) A single plasma treatment (hydrogen, nitrogen or argon plasma) mainly results in an increase of the surface roughness of the sputtered silver films and to an increase of the SERS activity. (e) Oxidation/reduction plasma treatment with various gases results in the formation of complex three-dimensional nanoporous silver structures with very strong SERS activity.

Thus controlled rf plasma treatment is a very interesting technique for the modification of surfaces as a number of different chemical species can be generated at low temperature in the gas phase, which can strongly interact with the exposed surface leading to distinct physical and chemical changes [63,76]. Therefore, it is interesting to investigate the influence of different kinds of rf plasmas on the surface of thin silver films, which are important substrates for SERS applications due to their great SERS enhancement factors. The question is whether plasma treatment can beneficially modify the thin silver films leading to an enhancement of their SERS activity or vice versa. Scheme 1 gives an overview of the steps performed in fabrication of the SERS substrates and their subsequent studies.

### As-sputtered silver films

**Figure 1:**
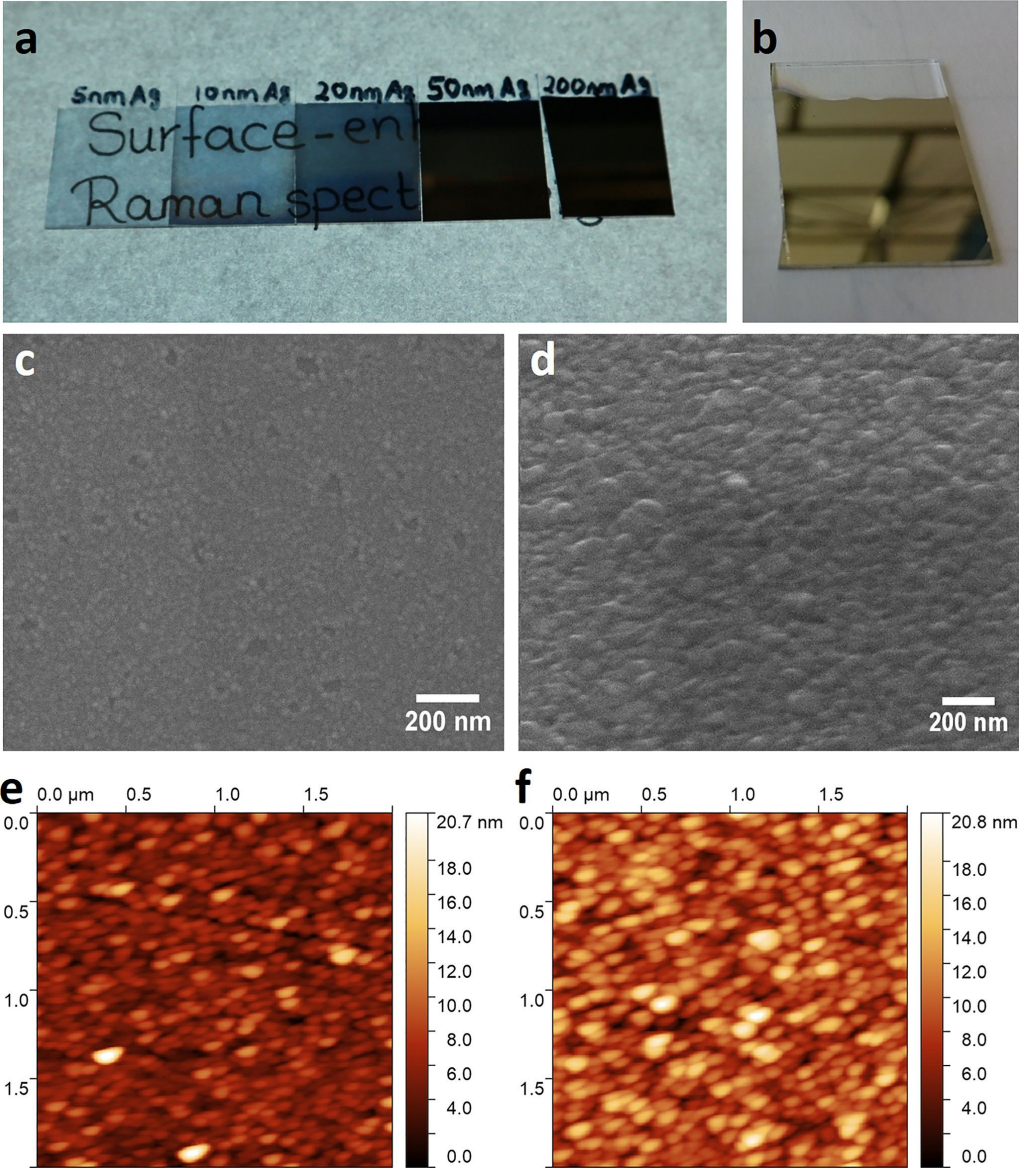
(a) Photograph of sputtered silver films on glass substrates with different thicknesses. From left to right: 5, 10, 20, 50 and 200 nm. (b) Photograph showing the mirror-like appearance of the 200 nm silver film. SEM images of (c) 10 nm and (d) 200 nm silver films. AFM images of (e) 10 nm (*R*_q_ = 2.02 nm) and (f) 200 nm (*R*_q_ = 2.79 nm) sputtered silver films.

Different thicknesses of silver have been sputtered on cleaned glass substrates. At very low thicknesses up to about 20 nm a transparent silver film could be observed, which became increasingly reflective and finally non-transparent with increasing thickness (Figure 1a,b).

Low-magnification SEM images of freshly sputtered silver films (10 and 200 nm) show no significant structural features, which confirms the uniformity of the sputtered silver films. In the 10 nm thick silver film small grains adjacent to each other together with some voids are observed due island formation during sputtering (Figure 1c,d) [77]. The granular morphology could be advantageous for the formation of hot spots resulting in an efficient SERS activity of the silver film. For the 200 nm thick silver film the coalescence of the adjacent silver clusters formed in the early stages of the sputtering deposition is visible, leading to a continuous silver film having a visible surface roughness. The sputter deposition yields silver films with a certain surface roughness (Figure 1e,f), which appears to be useful for SERS applications. However, in order to increase this initial surface roughness even more and to create further potential hot spots for increased SERS performance, systematic rf plasma treatments under different conditions were investigated.

### Formation of hydrogen plasma treated silver films

**Figure 2:**
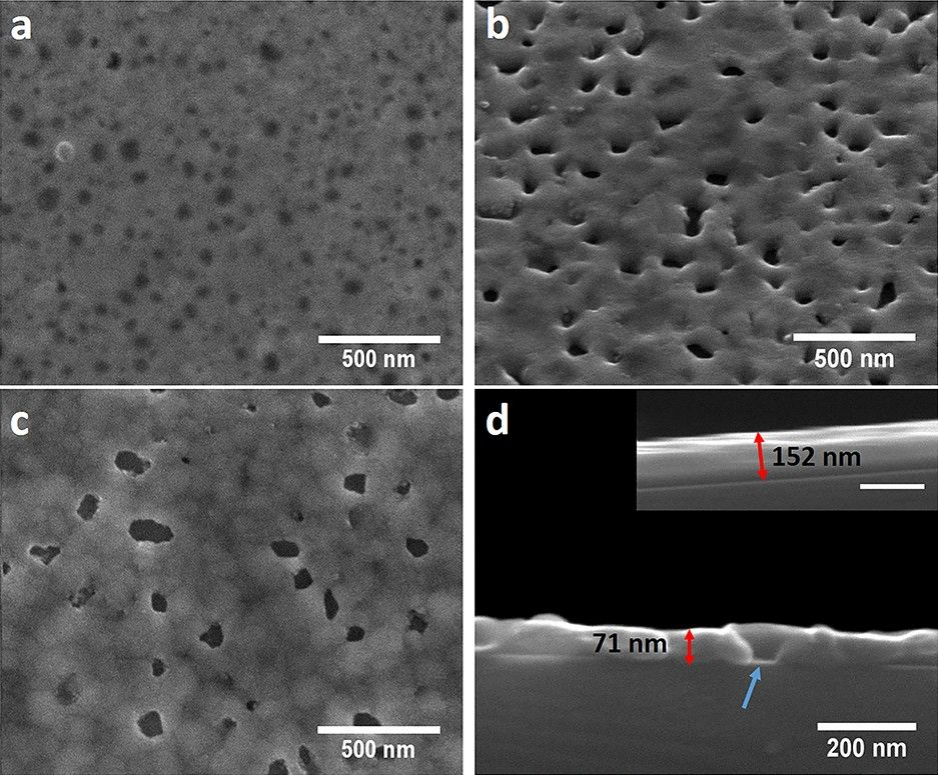
SEM image of a 200 nm sputtered silver film treated with hydrogen plasma (g12-p200) for (a) 5 min, (b) 30 min (taken at 45° tilt) and (c) 45 min at 100000× magnification showing the formation of etched holes and increasing grain structure of the silver film. (d) Cross-sectional SEM image of a 200 nm sputtered silver film after 30 min of hydrogen plasma treatment (g12-p200) depicting the actual lowered thickness of the hydrogen plasma-treated silver films compared to the as-sputtered silver film seen in the inset (scale bar: 200 nm). The blue arrow indicates a hole in the hydrogen plasma treated silver film.

Employing hydrogen plasma treatment (200 W) using a gas flow rate of 12 sccm results in a chamber pressure of 0.38 mbar. The maximum treatment time was set to 2 min for the ultrathin 10 nm silver films. For the 200 nm silver hydrogen plasma treatment was performed for 5, 15, 30 and 45 min. The SEM images in Figure 2 and Figure S1 (Supporting Information File 1) reveal black spots on the silver surface that become darker and increase in size with increasing hydrogen plasma treatment time. These areas show the formation of holes in the silver film which deepen and increase in size with increasing hydrogen plasma treatment time. Using AFM (Figure S2, Supporting Information File 1) it shows that surface roughness increases from 2.02 nm for an untreated silver film to 5.03 nm for 5 min, 6.29 nm for 15 min and 6.85 nm for 45 min hydrogen plasma treatment time. The relatively small increase in surface roughness when going from 15 to 45 min can be explained by the fact that with increasing hydrogen plasma treatment time the etched holes become deeper but at the same time silver is etched away from the surface leading to a decrease in the overall film thickness. Thus, the overall peak-to-valley distance will not change much. A cross-sectional SEM of the silver films before and after hydrogen plasma treatment (Figure 2d) clearly reveals the presence of holes as well as a decrease in the silver film thickness from about 152 nm to about 71 nm supporting the claim that the silver is effectively etched away by hydrogen plasma with an average etch rate of 2.7 nm/min. Etching of silver films by means of hydrogen plasma in an inductively coupled plasma system has also been previously observed and the reasons for this etching could be ion bombardment leading to physical sputtering together with chemical etching for which the formation of a silver dihydride anion (AgH_2_^−^) as etching product has been suggested as it is more stable than silver hydride [78]. As can be seen in the top SEM images (Figure 2a–c) and in the cross-sectional SEM images (Figure 2d) holes are formed in the silver film, which increase in size and depth with increasing hydrogen plasma treatment time. The formation of the holes can be mainly attributed to physical sputtering of silver due to ion bombardment. But at the same time there is a homogenous decrease in the thickness of the silver film, which would point to a chemical etching process that takes place over the whole area exposed to the plasma. Other samples treated with hydrogen plasma using different plasma gas flow conditions and plasma power show the ability of this process for tuning the surface morphology and roughness further (Figure S3, Supporting Information File 1).

### Formation of nitrogen plasma treated silver films

**Figure 3:**
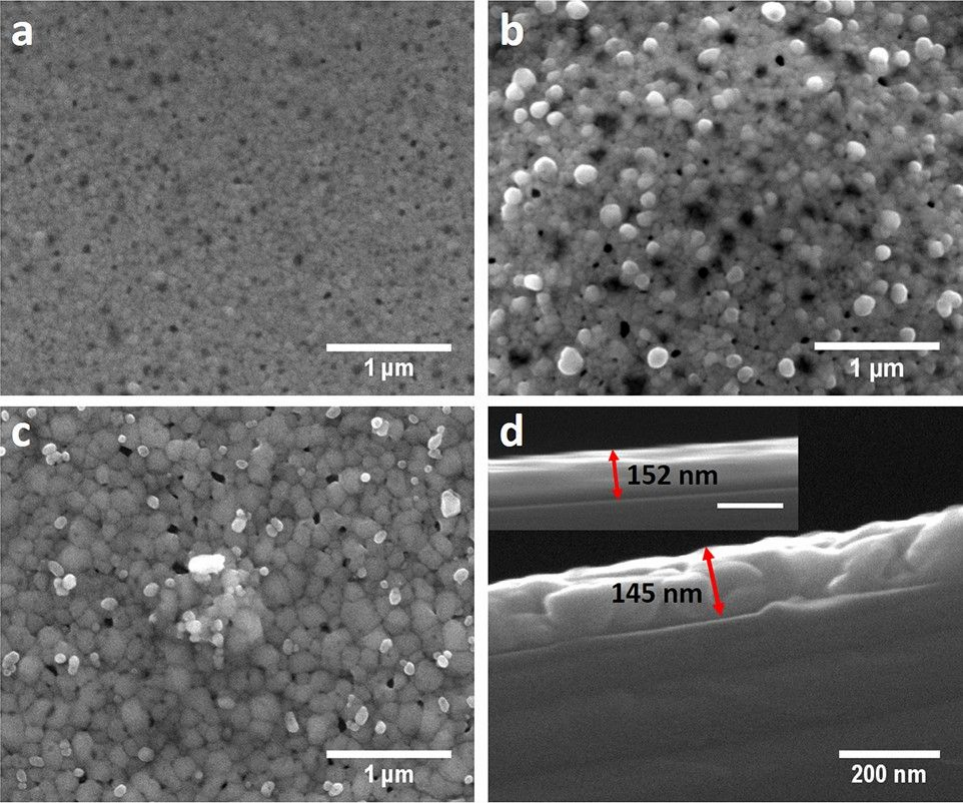
SEM image of a 200 nm sputtered silver film treated with nitrogen plasma (g12-p200) for (a) 10 min, (b) 30 min and (c) 60 min at 50000× magnification. (d) Cross-sectional SEM image of 200 nm sputtered silver film treated with nitrogen plasma (g12-p200) for 30 min showing the actual thickness of the prepared silver film after nitrogen plasma treatment compared to the as-sputtered silver film seen in the inset (scale bar is 200 nm).

Treatment of the 200 nm thick silver films with a nitrogen rf plasma does create a groove-like morphology, which transforms over time (10–60 min) into deeper voids (Figure 3 and Figure S4, Supporting Information File 1). This comes along with an increasing particle formation with increasing treatment time (from 10 to 30 min). Furthermore, a faceting of the silver film underlying the formed nanoparticles is visible, which becomes more prominent with increasing nitrogen plasma treatment time from 10 to 60 min (Figure 3a–c). For the ultrathin 10 nm film as well as for the 50 nm film, this granular and facetted surface can be also observed depending on the nitrogen plasma treatment time (Figure S5, Supporting Information File 1). AFM analysis (Figure S6, Supporting Information File 1) revealed a significant increase in the surface roughness of the 200 nm silver films treated with nitrogen plasma, depending on the treatment time. After 10 min of nitrogen plasma treatment, the surface roughness *R*_q_ was 5.12 nm and reached 23.1 nm after 60 min of treatment. The formation of grooves, which is also observed after hydrogen plasma treatment, is a result of the physical sputtering effect of the silver surface. However, the formation of a particulate morphology and the faceting of the silver surface is best explained by an anisotropic etching or a restructuring process of the silver surface by reactive nitrogen plasma species such as N_2_^+^, N_2_^*^ (excited nitrogen), N^•+^ and N^•^ (nitrogen radicals) [79]. The faceting of the silver surface is depends on the plasma treatment time when using varying nitrogen plasma treatment parameters and leads to different structural characteristics with respect to the surface roughness (Figure S7 and Figure S8, Supporting Information File 1).

A cross-sectional SEM image of a nitrogen plasma treated silver film (Figure 3d) shows a slight decrease in film thickness compared to untreated silver films, which indicates minor etching by the nitrogen plasma mainly due to ion bombardment. A reasonable explanation for the restructuring of the silver surface, which leads to particle formation and surface faceting could be the in situ formation of silver nitride, which directly decomposes to metallic silver and nitrogen gas [80]. This dynamic process of incorporation of nitrogen followed by its extrusion could lead to a granular structure of the silver surface.

### Materials characterization of hydrogen and nitrogen plasma treated silver films

**Figure 4:**
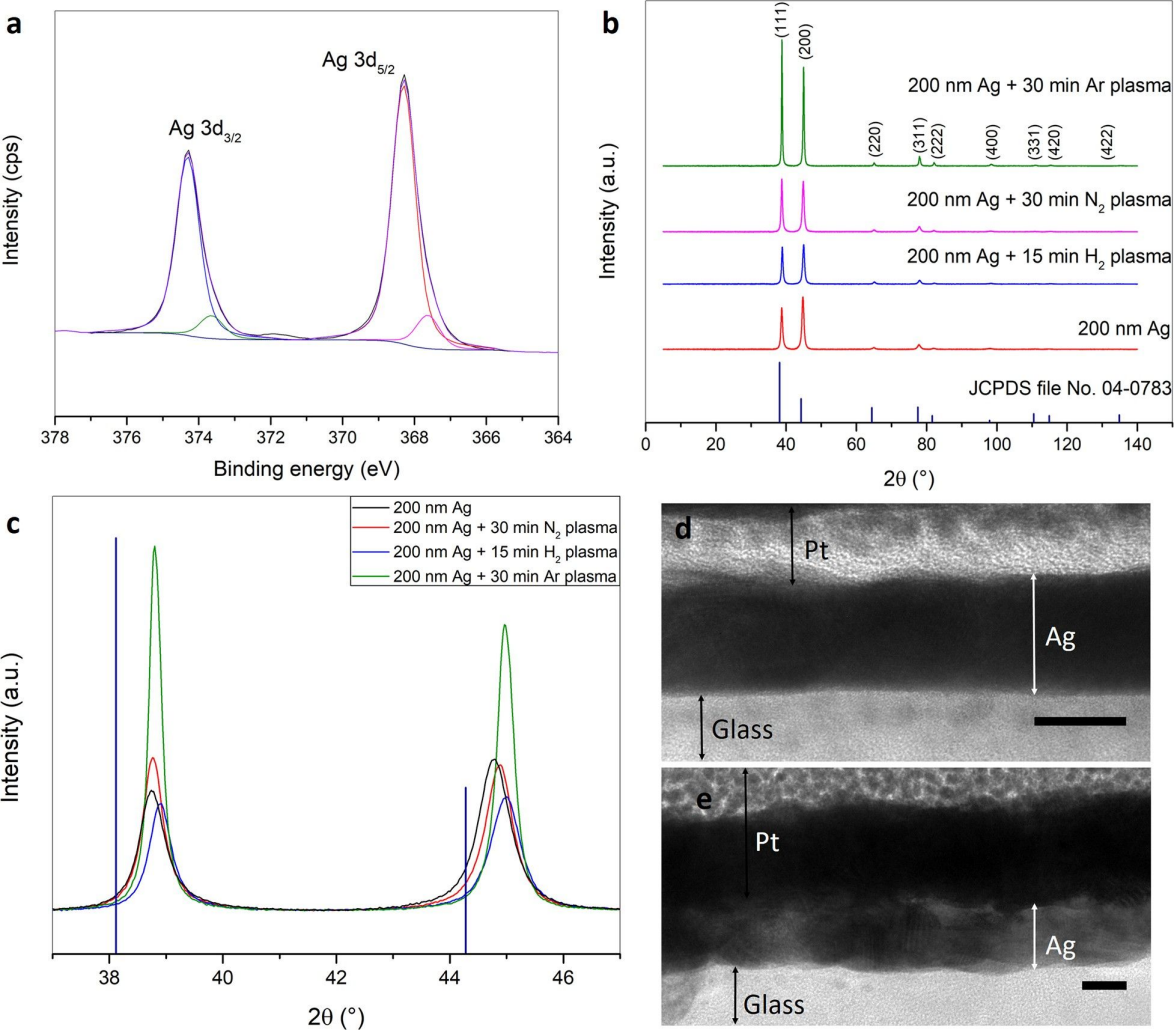
(a) Deconvoluted XPS Ag 3d spectrum for 200 nm Ag + 30 min nitrogen plasma treatment (g12-p200). (b, c) XRD of 200 nm Ag, 200 nm Ag + 15 min hydrogen plasma treatment (g12-p200), 200 nm Ag + 30 min nitrogen plasma treatment (g12-p200), 200 nm Ag + 30 min argon plasma (g16.7-p200) and their comparison with standard XRD pattern for silver as reference (JCPDS file No. 04-0783). Cross-sectional TEM (FIB) images of (d) an as-sputtered 10 nm silver film and (e) 10 nm silver + 5 min nitrogen plasma treatment (g12-p200). Scale bar: 10 nm.

Neither XPS analysis nor Auger spectroscopy could detect the presence of nitrogen in the nitrogen plasma treated samples (Figure 4a and Figures S9–S11 and Table S1 and Table S2, Supporting Information File 1). Even depth profiling using Auger spectroscopy did not reveal any traces of nitrogen (Figure S12, Supporting Information File 1). For further insight, the XPS spectra of untreated sputtered silver, hydrogen plasma treated silver and nitrogen plasma treated silver was compared. The comparison of the Ag 3d peaks obtained for the three samples shows that there is a slight broadening in the direction of lower binding energies for nitrogen plasma treated silver (Figure 4a and Figure S9a, Supporting Information File 1). Only for the nitrogen plasma treated silver film, a second small peak at 367.63 eV can be fitted besides the main peak at 368.30 eV in the silver 3d peaks (Figure 4a and Figure S9b,c, Supporting Information File 1). Auger spectroscopy of the nitrogen plasma treated silver film shows a typical spectrum in which only the elements silver, carbon, oxygen, and no nitrogen, can be found (Figure S9d, Supporting Information File 1). The reference position of the different elements [81] was inserted and there is a small shift of the silver peak relative to the reference value. The presence of carbon and oxygen, which were also detected by XPS, is due to adventitious carbon and chemisorbed oxygen as the sample was in contact with the atmosphere before the measurement. Although XPS and Auger measurements could not detect nitrogen functionalization on the metal surface, these techniques do reveal that nitrogen plasma treated silver films differ slightly from the as-sputtered films and from the hydrogen plasma treated films. This indicates, together with the different surface structure, a dedicated influence of the nitrogen plasma in addition to the impact of nitrogen ions on the silver surface.

XRD analysis of the 200 nm as-sputtered silver film as well as hydrogen, nitrogen and argon plasma treated silver films of this thickness show a dependence of the significant and most prominent reflexes on the film texture (Figure 4b). As can be seen from Figure 4b and Figure 4c the preferential orientation of the crystallites is different from that of bulk silver (JCPDS file No. 04-0783). In case of the as-sputtered silver film the crystallites show a preferential (200) orientation. After plasma treatment a gradual change of the preferential orientation can be observed. After argon plasma treatment the preferential orientation completely changes to (111). At the same time the peak width and the positions of the (111) and (200) reflexes after different plasma treatments slightly change when compared to those of as-sputtered silver (Figure 4c). This indicates changes in the lattice parameters and thus a change in the macro- and microstrains present in the silver film. All these changes can be explained by the energy transferred to the silver atoms through the impact of high energy ions and electrons on the silver surface during plasma treatment. This leads to an increased mobility of the silver atoms, which either rearrange on the surface by displacement or are completely removed through sputtering.

A comparison of the cross-sectional TEM (FIB) images of an as-sputtered 10 nm silver film with a 10 nm silver film treated with nitrogen plasma (g12-p200) for 5 min shows a decrease in the silver film density indicated by a contrast change through which the boundaries of the different crystallites become visible (Figure 4d,e). At the same time, a morphological change of the interface of silver to the substrate is observed. It shows an increased roughness indicating that the plasma treatment affects the complete thickness of the silver film down to the substrate interface.

### Argon plasma treated silver films

**Figure 5:**
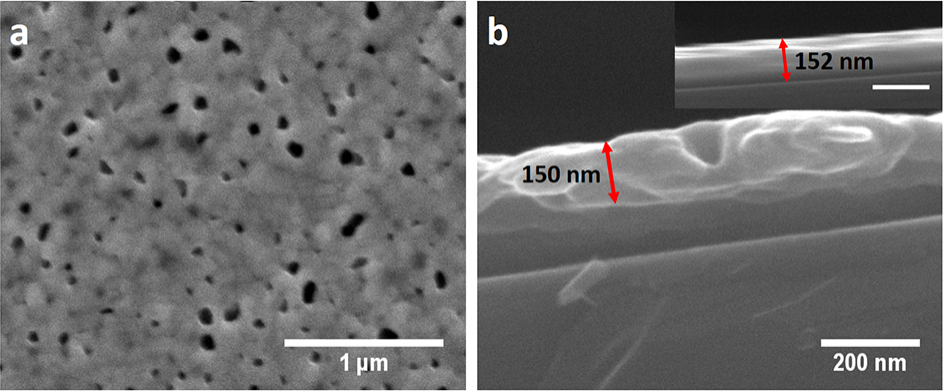
(a) SEM image of 200 nm sputtered silver film treated with argon plasma (g16.7-p200) for 30 min. (b) Cross-sectional SEM image of 200 nm sputtered silver film treated with argon plasma (g16.7-p200) for 30 min showing the actual thickness after argon plasma treatment compared to the as-sputtered silver film shown in the inset (scale bar: 200 nm).

Compared to the nitrogen plasma treated silver films, argon plasma treatment of the sputtered silver films resulted in the distinct formation of voids similar to those in the silver films treated with hydrogen plasma. But in contrast to the hydrogen plasma treated films no depth etching effect was observed in the cross-sectional SEM (Figure 5).

### Plasma treated silver films under oxidation/reduction conditions

Oxygen rf plasmas are well known to efficiently oxidize metallic silver films resulting in granular and nanoporous silver oxide films [73]. For any application in SERS a subsequent reduction to metallic silver is necessary. However, the advantage of this oxidation process is its capability to completely change the morphology of the silver surface converting the originally flat silver surface into a three-dimensional nanoporous surface. The use of hydrogen, nitrogen or even argon plasma as a dry reducing agent would provide an attractive alternative to the often used wet chemical process [75] for obtaining efficient SERS substrates from previously oxidized silver films.

**Figure 6:**
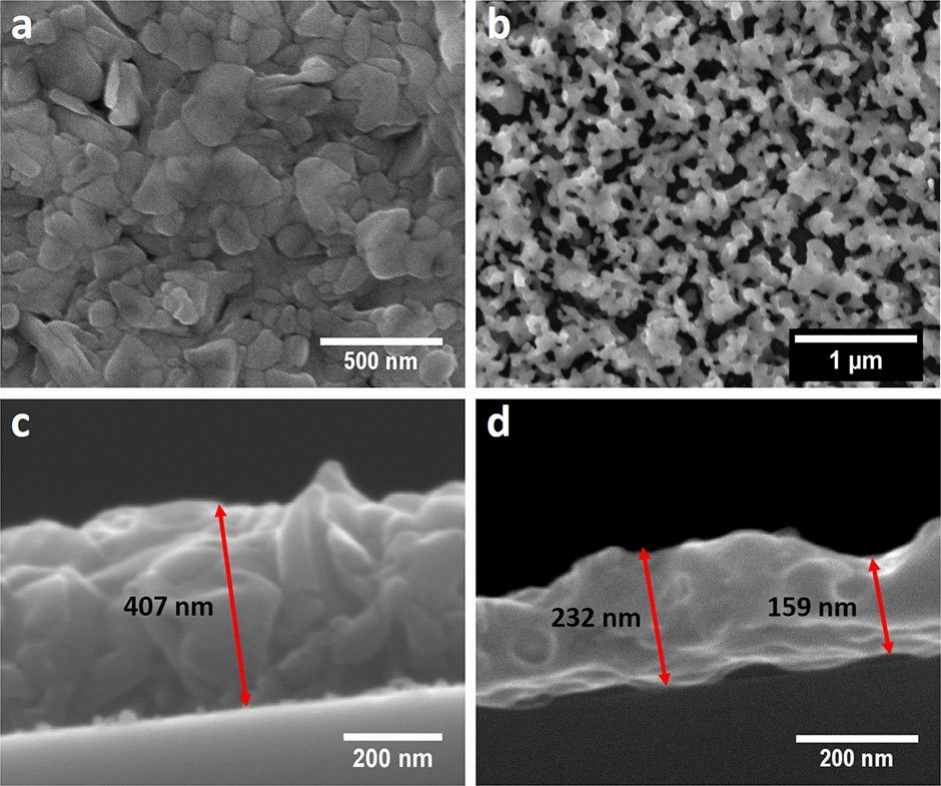
SEM images of a 200 nm sputtered silver film treated with (a) oxygen plasma (g12-p200) for 15 min and (b) after reducing the oxidized silver film with hydrogen plasma (g12-p200) for 20 min. Cross-sectional SEM image of 200 nm sputtered silver film treated with (c) oxygen plasma (g12-p200) for 15 min and (d) after reducing the oxidized silver film with hydrogen plasma (g12-p200) for 20 min showing the actual thickness of the prepared silver film after plasma treatment.

A 200 nm sputtered silver film was treated with oxygen plasma at a power of 200 W and a chamber pressure of 0.8 mbar for 15 min. The reduction of the oxidized silver film was performed with a hydrogen plasma at a power of 200 W and a chamber pressure of 0.38 mbar for 20 min in order to ensure complete reduction of the silver oxide film. Oxidation of sputtered silver films with oxygen plasma yields a polycrystalline silver oxide film with distinct grain boundaries (Figure 6a). After reduction of the silver oxide film to silver a highly porous structure is formed (Figure 6b). At the same time the drastic increase in film thickness compared to the as-sputtered silver film is observed (Figure 6c). The measured film thickness from the cross-sectional SEM for the oxidized silver film is about 407 nm, which is about 2.7 times the film thickness measured for the sputtered silver film. This large increase in film thickness is obviously due to the formation of silver oxide during the oxygen plasma treatment as a mixture of Ag_2_O and AgO [82]. The incorporation of oxygen into the silver lattice leads to a volume expansion and a change in film morphology. The subsequent reduction of this silver oxide film with the hydrogen plasma results in a subsequent film shrinkage back to nearly half of its former value (159–232 nm; Figure 6d) [83–84]. Due to the volume shrinkage the film porosity increases significantly.

Changing plasma treatment time, chamber pressure and plasma power of the oxygen and hydrogen plasmas allows for a further tuning of the morphology of the nanoporous silver films (Figure S13, Supporting Information File 1). Longer treatment with hydrogen plasma can lead to further etching of the pores and the surface after completion of the reduction, while a longer oxygen plasma treatment leads to smaller pores.

An AFM study of the 200 nm oxidized/reduced silver films proves the drastic increase in surface roughness with increasing oxygen and hydrogen plasma treatment times and plasma power (Figure S14, Supporting Information File 1). A surface roughness *R*_q_ of 38.4 nm was obtained for a 200 nm sputtered silver film treated for 20 min with oxygen plasma (g12-p200) followed by another 20 min of hydrogen plasma (g12-p200) treatment.

**Figure 7:**
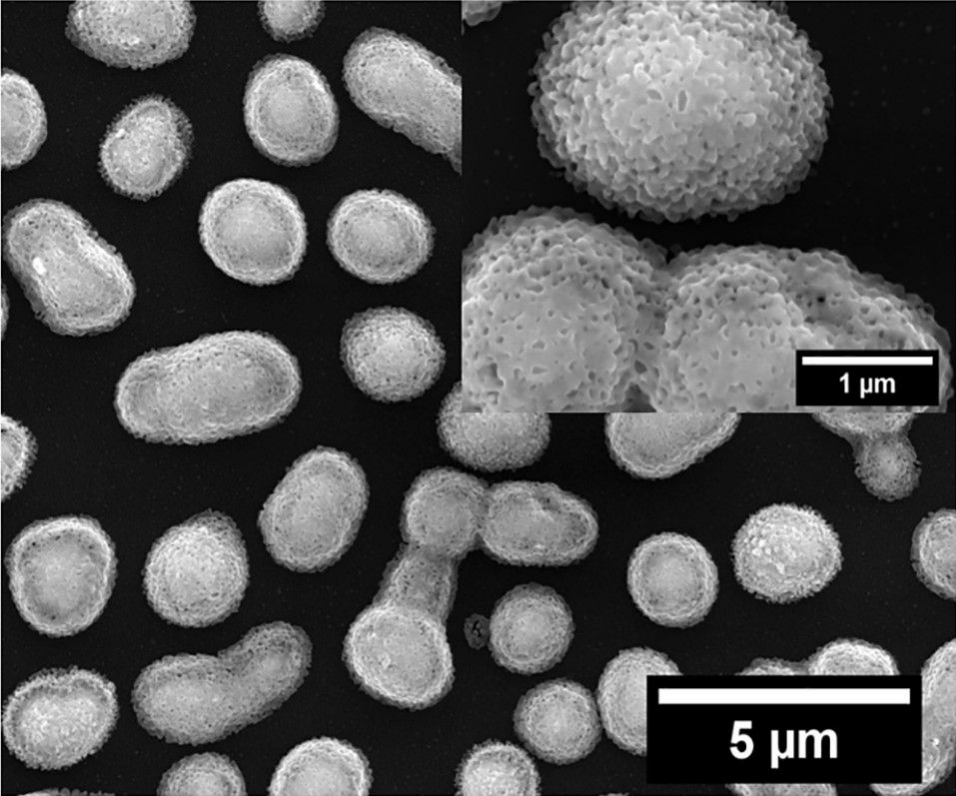
SEM image (10000× magnification) of a 200 nm sputtered silver film heated at 400 °C for 15 min followed by plasma treatment with oxygen plasma (g12-p200) for 10 min and reduction with hydrogen plasma (g12-p200) for another 10 min. The inset shows particles at 50000× magnification.

If however, the as-sputtered silver film is heated before plasma treatment to 400 °C for 15 min, dewetting of the continuous silver film into isolated silver particles occurs [85]. Oxidation of these particles by oxygen plasma followed by reduction by hydrogen plasma results in nanoporous particles with a spongy morphology (“silver nanosponges”, Figure 7).

In addition, argon plasma was tested as an alternative to hydrogen plasma for the reduction of the silver oxide films obtained after oxygen plasma treatment. In this case the reduction process mainly depends on the electron density of the used plasma. The reduction of silver oxide is facilitated by plasma treatment due to the presence of high-energy UV photons that can easily break the silver–oxygen bond [78,86].

**Figure 8:**
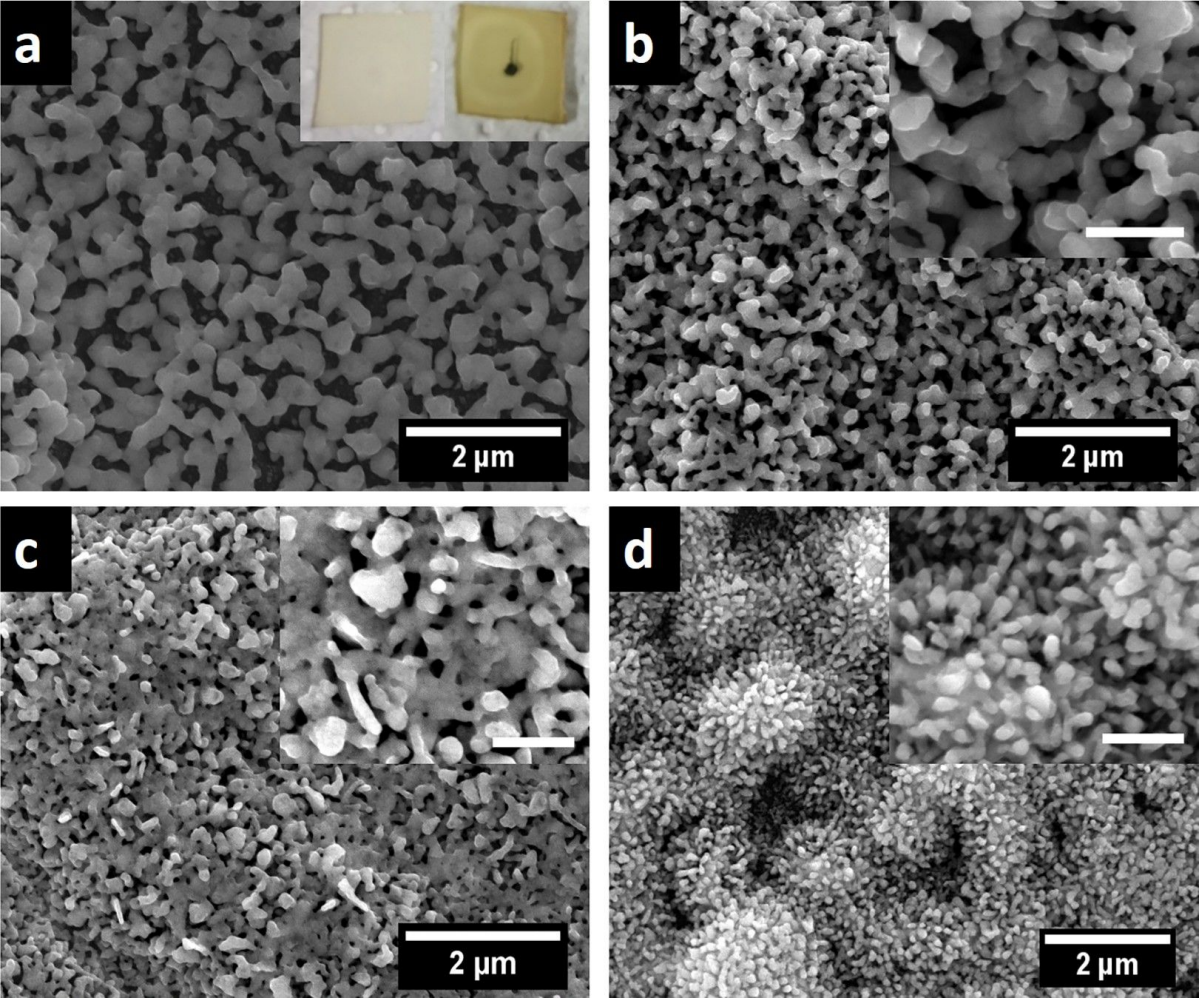
SEM images of a 200 nm sputtered silver film treated with (a) a mixture of argon (8 sccm) and oxygen (4 sccm) in an rf plasma (200 W) for 15 min followed by reduction using a pure argon plasma (g16.7-p200) for 15 min; the inset displays a photograph of the top and the bottom (with black spot) side of the silver film on glass, (b) air plasma (g12-p200) for 15 min followed by reduction with argon plasma (g16.7-p200) for 20 min, (c) air plasma (g12-p200) for 30 min followed by reduction with argon plasma (g16.7-p200) for 20 min and (d) 200 nm sputtered silver film heated at 400 °C for 15 min followed by plasma treatment with air plasma (g12-p200) for 15 min and reduction with argon plasma (g16.7-p200) for another 15 min. The insets are at a 100000× magnification (scale bar: 500 nm).

Again a 200 nm silver film was first oxidized using an oxygen rf plasma for 15 min at a power of 200 W and 12 sccm oxygen flow rate (chamber pressure of 0.8 mbar). After that, the oxidized silver film was reduced using an argon plasma at a power of 200 W and an argon flow rate of 16.7 sccm (chamber pressure 1.5 mbar). The morphology of the nanoporous silver film is very similar to the one obtained from the reduction using hydrogen as reducing plasma (Figure S15, Supporting Information File 1). Further changes to the oxidation/reduction protocol were made in order to determine whether varying plasma conditions could lead to different 3D silver morphologies. Subsequently, a 200 nm sputtered silver film was treated with an rf plasma containing a mixture of argon and oxygen gas (8 sccm argon, 4 sccm oxygen gas flow at a power of 200 W for 15 min). Treatment of the so obtained oxidized silver film with pure argon rf plasma (argon flow rate 16.7 sccm, power 200 W, 15 min) results in a complex network of interconnected silver particles with large pore size (Figure 8a). This difference in morphology is already observed in the silver film oxidized by the argon/oxygen mixture when compared to the silver film oxidized by oxygen plasma alone (Figure S16a,d, Supporting Information File 1). The difference in the obtained morphologies can eventually be explained by a competing process of oxidation and reduction taking place during the plasma treatment with a mixture of argon and oxygen. Silver might be oxidized, followed by partial reduction both in a dynamic process. To proof this idea further, an rf plasma using air (78.08% N_2_, 20.95% O_2_ and 0.97% other gases) was used for the initial oxidation step (flow rate 12 sccm, 200 W for 15 min) followed by a reduction using argon plasma for 20 min (g16.7-p200). SEM reveals again a similar complex network of interconnected silver particles resembling a coral reef as found before when using the argon/oxygen mixture as oxidizing plasma (Figure 8b). Even the silver film oxidized by air plasma for 15 min and 30 min as well as the silver film oxidized by the argon/oxygen plasma mixture show different surface structures compared to the silver films oxidized by pure oxygen plasma (Figure S16, Supporting Information File 1). This indicates that the competing oxidation/reduction reactions might play an important role for the in situ structuring of the silver surface. Increasing the plasma treatment time when using an air plasma up to 30 min results in an intact porous silver film however with a dense arrangement of short protrusions on the silver particle surfaces (Figure 8c). This unique silver morphology might represent the preliminary stage for the coral reef structure obtained with longer argon plasma treatment relative to the plasma oxidation time. Heating an as-prepared silver film for 15 min at 400 °C followed by oxidation with air plasma (g12-p200) for 15 min and reduction with argon plasma (g16.7-p200) for another 15 min results in silver particles having short rod-like protrusions (Figure 8d). Finally, a reduction with nitrogen plasma was also performed on silver surfaces oxidized by oxygen and air plasma treatment and yielded similar results as those obtained with hydrogen or argon as reducing gases (Figure S17, Supporting Information File 1).

XRD analysis of the silver films after oxidation/reduction proves that the reduction to metallic silver is complete in all cases revealing only the reflexes of elemental silver without any traces of silver oxide (Figure S18, Supporting Information File 1). Comparison of the XRD of the silver nanostructures resulting from oxidation/reduction of silver films with as-sputtered silver films shows a variation of the preferential orientation of the silver crystallites in the films. With the systematic combination of oxidizing and reducing rf plasmas and a variation of the different plasma parameters, complex 3D silver nanostructures with tunable surface roughness and nanoporosity can be obtained.

### Optical behavior and surface wetting of differently plasma treated silver films

**Figure 9:**
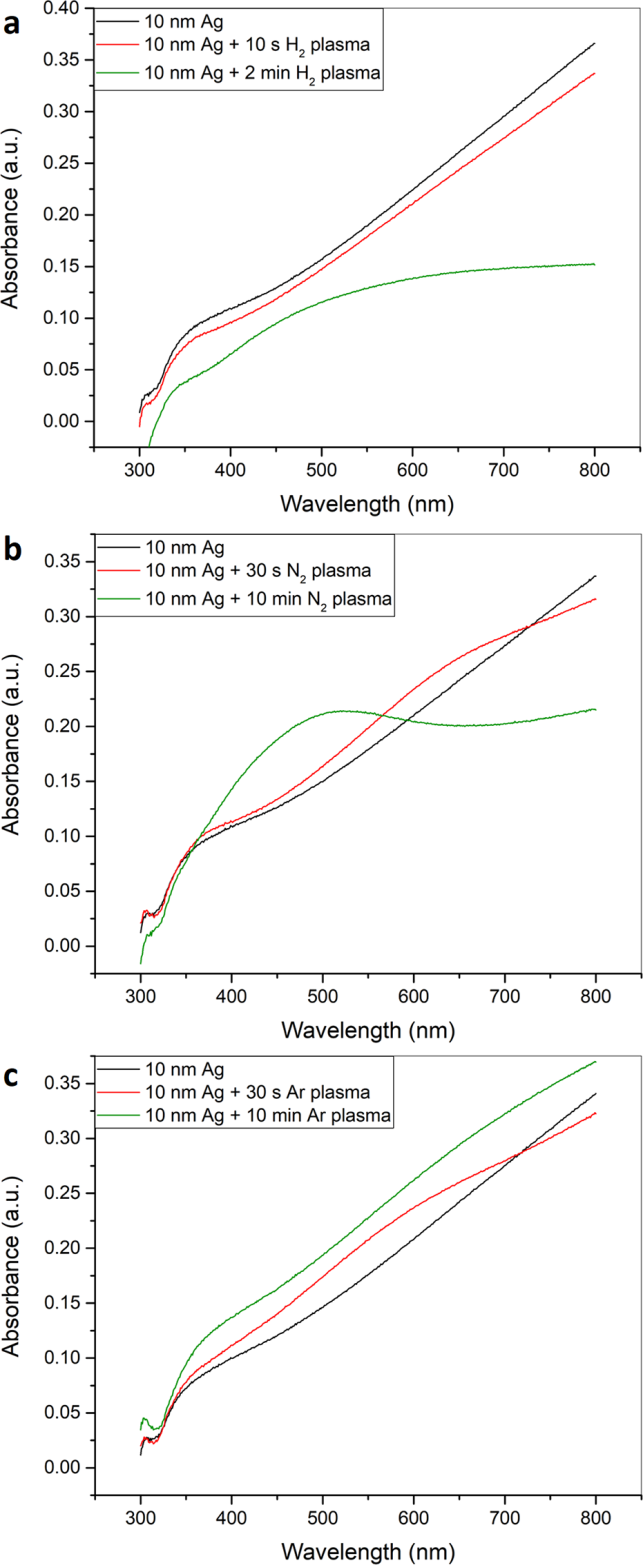
UV–vis spectra of 10 nm sputtered silver films treated with (a) hydrogen plasma, (b) nitrogen plasma and (c) argon plasma for different times.

Figure 9a shows the UV–vis absorption spectra obtained after two different hydrogen rf plasma treatment times in comparison to the silver film before plasma treatment. With increasing hydrogen plasma treatment time, the absorption decreases over the entire spectrum while the decrease is more prominent for longer wavelengths as can be seen for the 2 min hydrogen plasma treatment. This decrease in absorption in the hydrogen plasma treated films might be due to the etching of the silver film. For nitrogen plasma treatment the absorption spectra do not show an overall decrease in absorbance (Figure 9b). While the absorbance decreases for longer wavelengths it increases for shorter wavelengths resulting in the formation of an absorption shoulder due to surface plasmons. While this effect is hardly visible for 30 s nitrogen plasma treatment time at about 650 nm, it becomes much more prominent for 10 min nitrogen plasma treatment at about 500 nm. This indicates a restructuring of the film surface leading to the formation of particulate structures on the surface as has been proven by SEM. This restructuring might result in the emergence of localized surface plasmon resonances as they are well-known for silver nanoparticles. In case of argon plasma treatment (Figure 9c) increasing plasma treatment time results in an increase of absorption of the silver film over the whole UV–vis spectrum indicating some sort of densification of the silver film [87].

**Figure 10:**
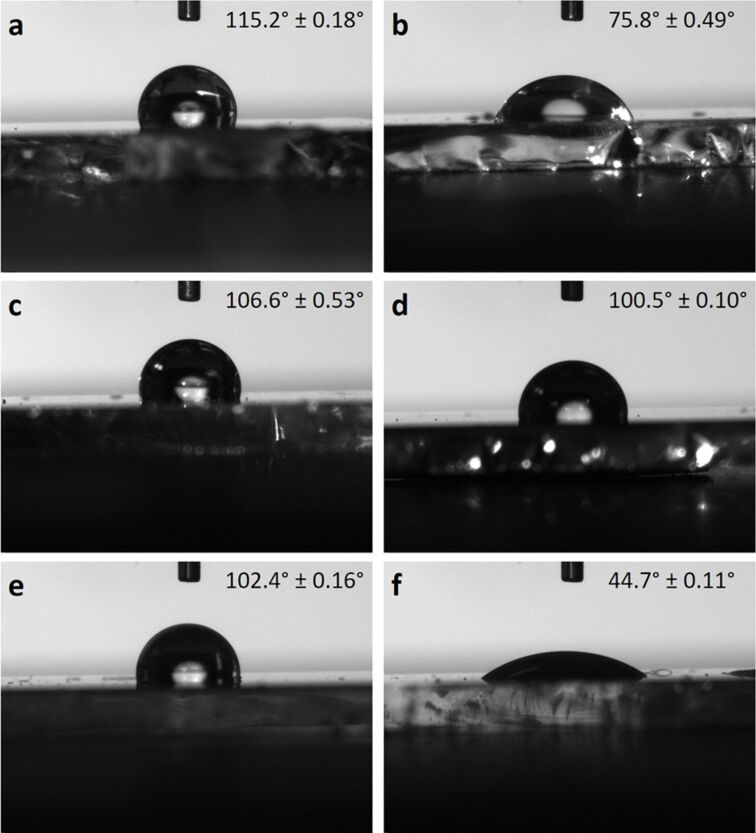
Water contact angle measurements on (a) 200 nm Ag, (b) 200 nm Ag + 15 min hydrogen plasma (g12-p200), (c) 200 nm Ag + 10 min nitrogen plasma (g12-p200), (d) 200 nm Ag + 60 min nitrogen plasma (g12-p200), (e) 200 nm Ag + 15 min oxygen plasma (g12-p200) + 20 min hydrogen plasma (g12-p200) and (f) 200 nm Ag + 15 min air plasma (g12-p200) + 20 min argon plasma (g16.7-p200).

The wetting behavior was analyzed by contact angle measurements of the plasma treated silver films (Figure 10). A change in surface wettability was obtained after the different plasma treatments. Obviously all rf plasma treatments lead to better wettabilities (water contact angles between 44.7° ± 0.11° and 106.6° ± 0.53°) when compared to the as-sputtered silver films (contact angle of 115.2° ± 0.18°). This enables a better contact between the analyte in aqueous solution and the SERS substrate. In all cases plasma treatment generates a more hydrophilic silver surface as compared to the as-sputtered silver films manifested by a decrease in the water contact angle. Especially after subsequent oxidation/reduction plasma treatments where nanoporous silver films are obtained, the silver films quickly absorb the droplets through capillary effects thus enabling the analyte to get in good contact with the silver surface.

### Evaluation of the SERS performance of rf-plasma treated silver films

The SERS effect for the different plasma treated silver films was evaluated in order to determine the influence of the different silver film morphologies obtained by plasma treatment on the SERS performance.

#### Effect of silver film thickness on SERS performance

**Figure 11:**
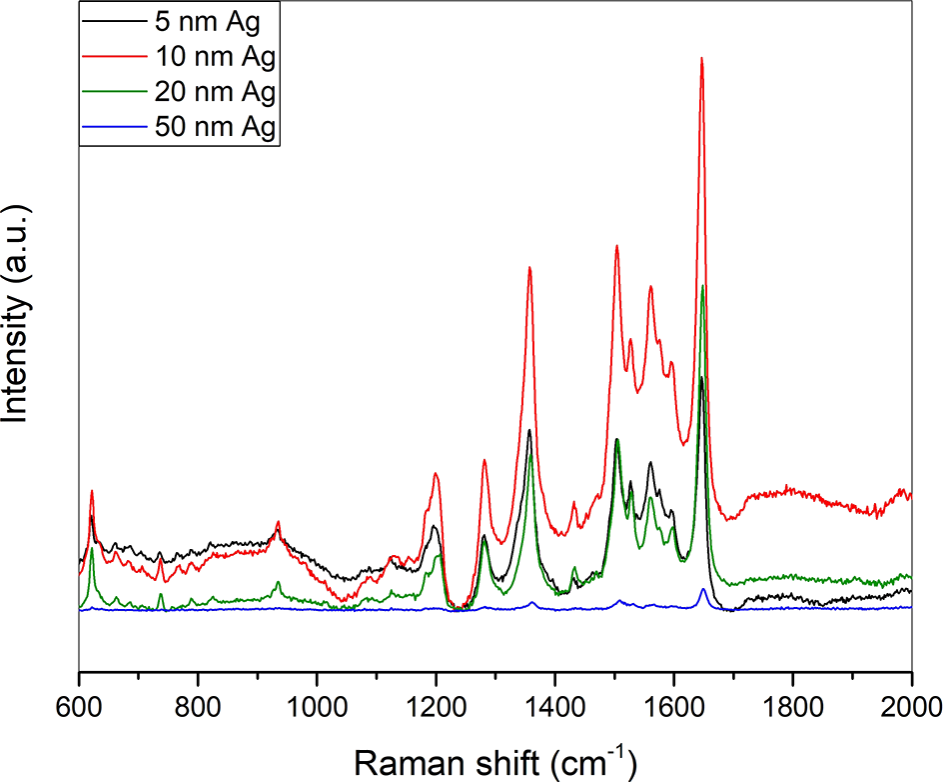
SERS spectra of 10^−6^ M RhB on sputtered silver films of different thicknesses.

First, the effect of thickness of the as-sputtered silver film on the SERS intensity was evaluated. 5 nm, 10 nm, 20 nm and 50 nm silver films obtained by sputtering were incubated for 30 min with a 10^−6^ M solution of RhB. SERS performance is increasing from 5 nm to 10 nm silver films and then decreases again with increasing thickness of the films (Figure 11). This can be attributed to the fact that during the initial stages of sputtering silver islands are formed on the substrate. The distance between these islands decreases with increasing sputtering time until the islands coalesce to form one continuous silver film [77]. Thus for the thinner sputtered silver film (10 nm) an optimal distance is generated between the silver islands leading to the presence of hot spots and an effective SERS enhancement. With increasing thickness of the films these hot spots disappear leading to a marked decrease in the SERS performance. The 10 nm sputtered film and 200 nm sputtered film exhibit similar roughness values (AFM, insets of Figure 2c and Figure 2d) despite the huge difference in SERS performance. This underlines the importance of the generation of hot spots for the evolution of high-intensity SERS spectra.

#### Effect of single plasma treatment on SERS performance

**Figure 12:**
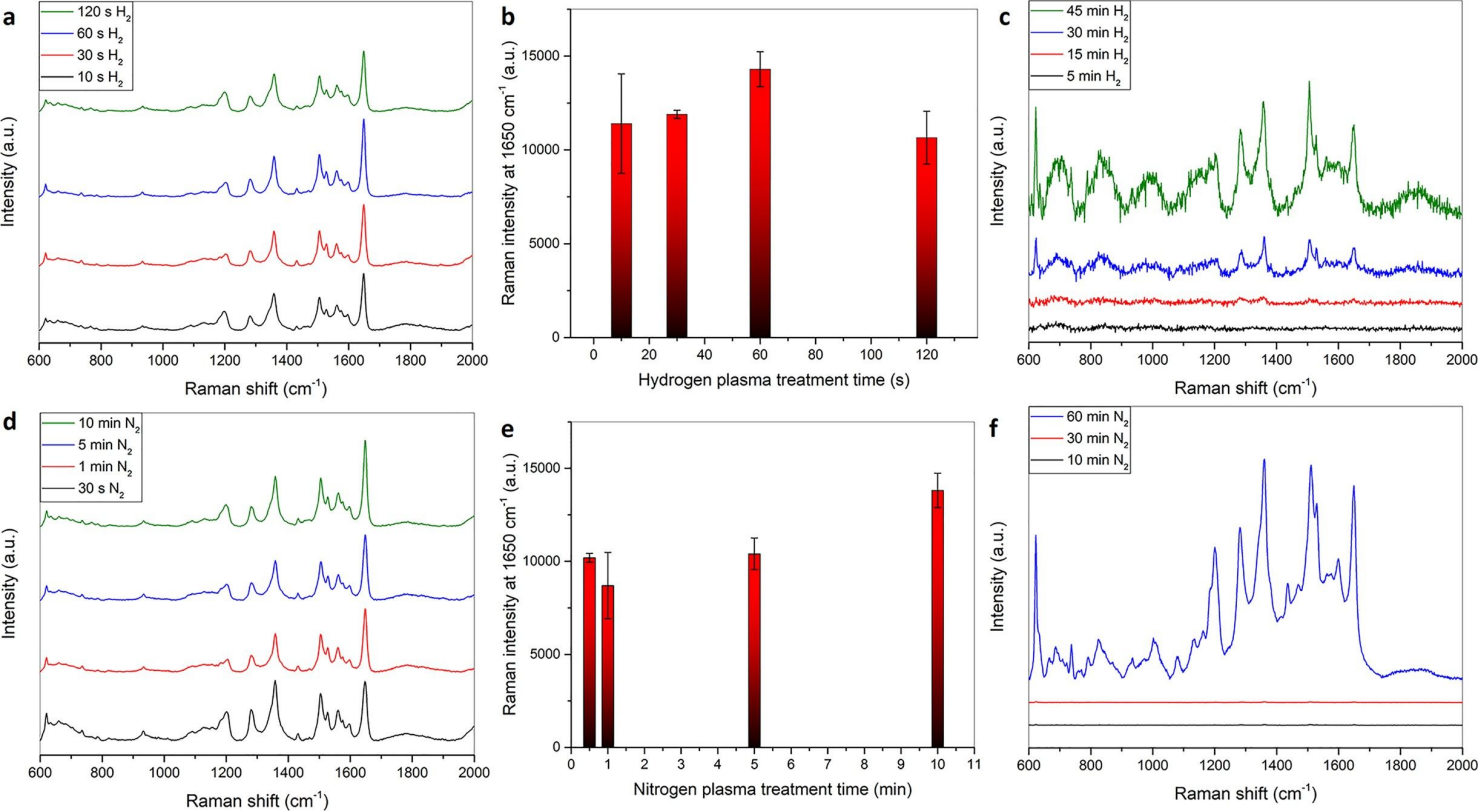
(a) SERS spectra of 10^−6^ M RhB deposited on a 10 nm silver film treated with hydrogen plasma (g12-p200) using a 514.5 nm laser. (b) Comparison of the average Raman intensities at 1650 cm^−1^. (c) SERS spectra of 10^−6^ M RhB on 200 nm Ag treated with hydrogen plasma (g12-p200) using a 632.8 nm laser. (d) SERS spectra of 10^−6^ M RhB on a 10 nm silver film treated with nitrogen plasma (g12-p200) using a 514.5 nm laser. (e) Comparison of the average Raman intensities at 1650 cm^−1^. (f) SERS spectra of 10^−6^ M RhB on a 200 nm silver flm treated with nitrogen plasma (g12-p200) using a 632.8 nm laser.

Hydrogen plasma treatment (g12-p200) on 10 nm silver films shows an increase in the SERS intensity with increasing treatment time until 60 s (Figure 12a,b). After longer hydrogen plasma treatment times the SERS intensity decreases, and after about five minutes of hydrogen plasma treatment the silver film has vanished from the glass substrate. This also explains why the SERS intensity decreases with increasing hydrogen plasma treatment time. As earlier corroborated by AFM and SEM (Figure 2 and Figures S1–S3, Supporting Information File 1), the hydrogen plasma increases the surface roughness but at the same time etches the silver layer. Therefore, the SERS intensity increases with very short hydrogen plasma treatment and then rapidly decreases for treatment times over 60 s for the ultrathin 10 nm silver film. With 200 nm silver films the same behavior could be observed (Figure 12c). With increasing hydrogen plasma treatment time, the SERS intensity increases, which is attributed to the increase in surface roughness (see AFM, Figure S2, Supporting Information File 1).

Nitrogen plasma treatment of the silver films (g12-p200) also shows an increase of the SERS intensity (Figure 12d–f). Interestingly, there is a sharp increase in the SERS intensity from 30 to 60 min nitrogen plasma treatment of the 200 nm silver film, which cannot be explained just by the increase in surface roughness. However, it could be due to a combined effect of increased surface roughness and a matching of the localized surface plasmon resonance with the employed excitation wavelength.

**Figure 13:**
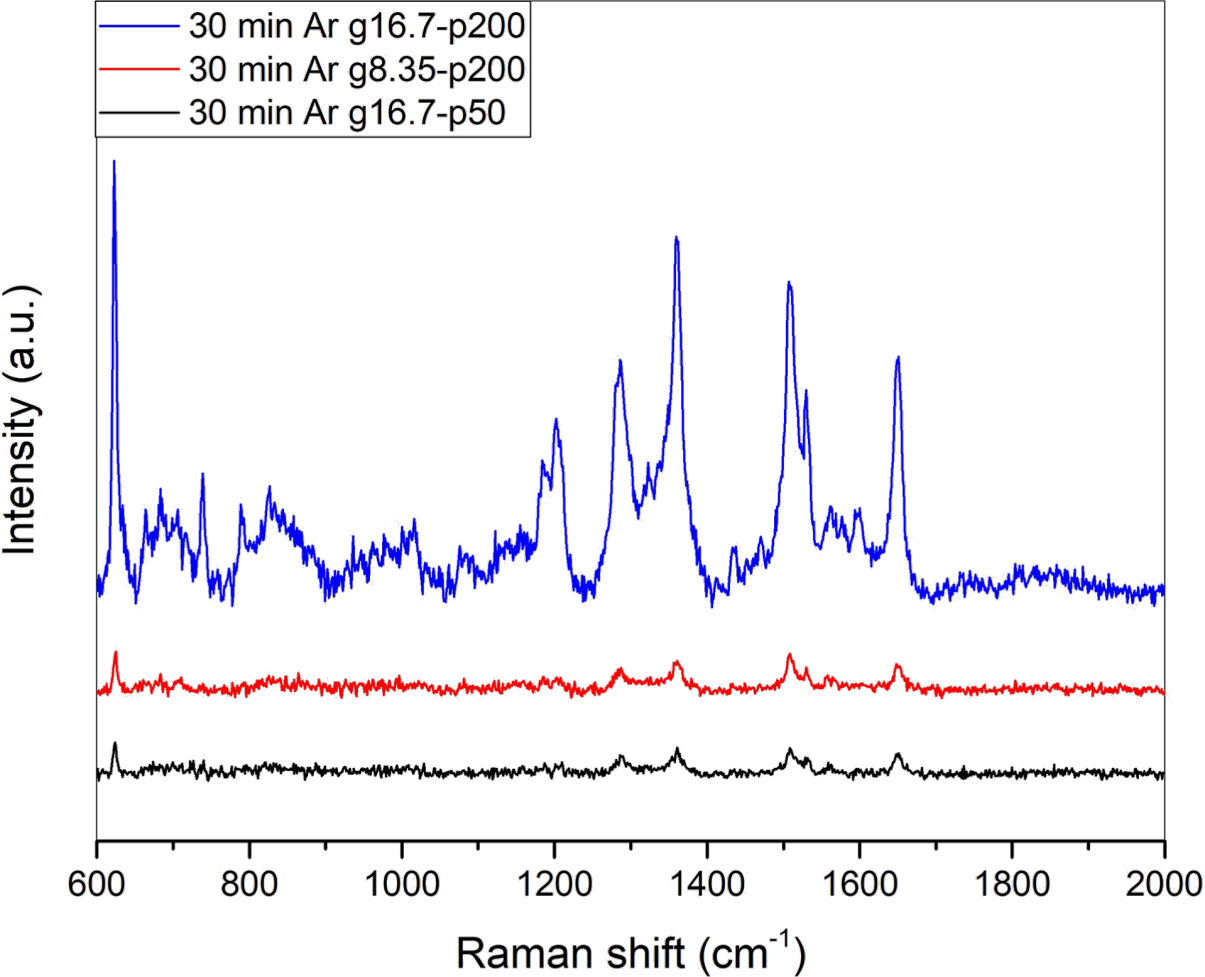
SERS spectra of 10^−6^ M RhB on a 200 nm silver film treated with argon plasma for 30 min using different parameters under 632.8 nm laser excitation.

Argon plasma treatment also leads to an increase in the surface roughness and an increase in SERS intensity. Changing the conditions of the plasma treatment leads to the ability to adjust the surface roughness and thus a change in SERS spectra while keeping the treatment time constant (Figure 13).

#### Effect of oxidation/reduction plasma treatment on SERS performance

**Figure 14:**
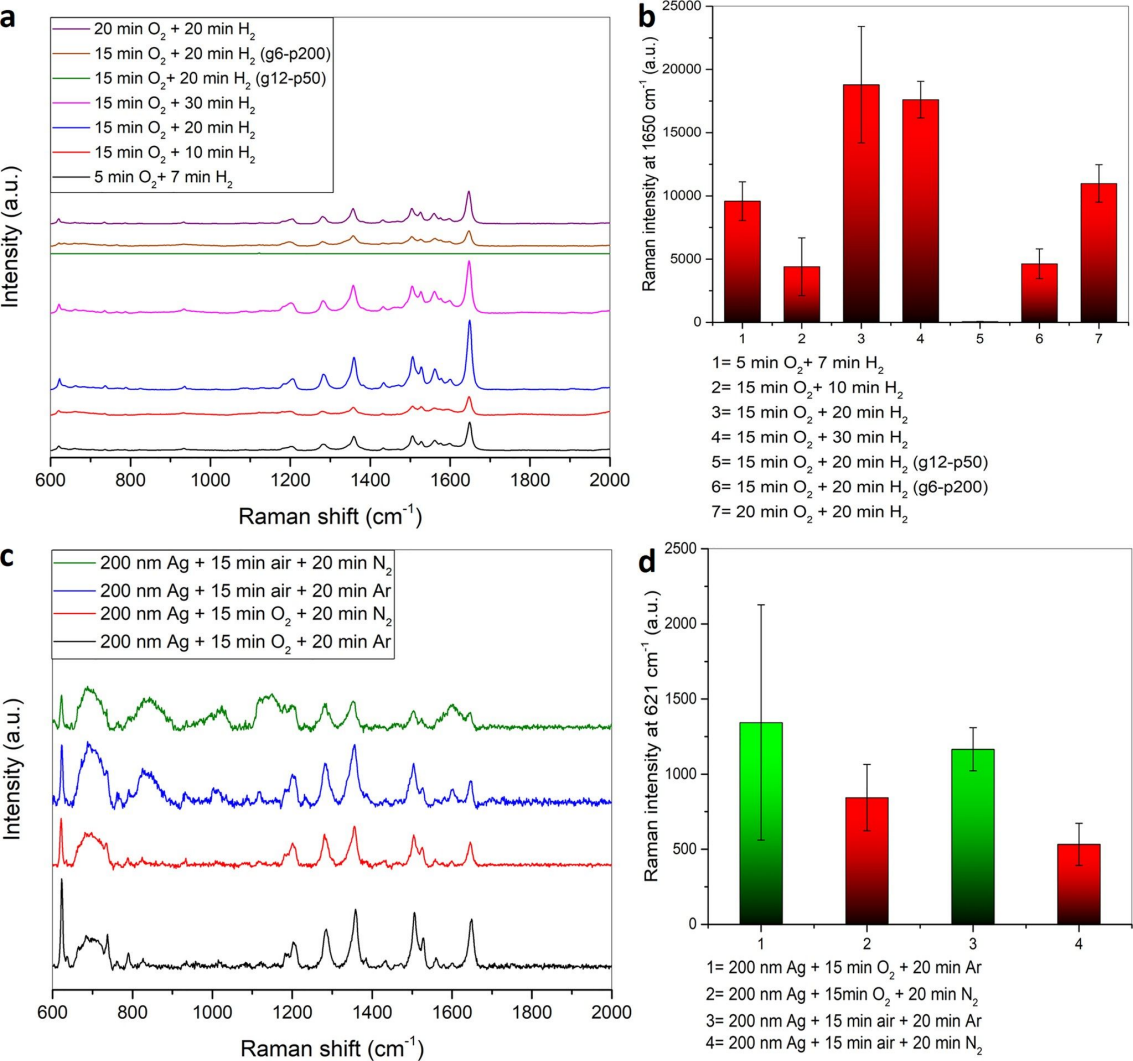
(a) SERS spectra of 10^−6^ M RhB on a 200 nm silver film treated with different times and parameters of oxygen and hydrogen plasma using 514.5 nm laser as excitation source. (b) Comparison of the average Raman intensities at 1650 cm^−1^. (c) SERS spectra of 10^−6^ M RhB on a 200 nm silver film treated with different oxidizing and reducing plasmas using 632.8 nm laser. (d) Comparison of the average Raman intensities at 621 cm^−1^.

To obtain more complex silver surface morphologies, oxidation followed by reduction of the as-deposited silver films was performed and studied for their SERS activity. As shown in Figure 14a and Figure 14b, different plasma treatment times for oxygen and hydrogen plasma as well as different plasma conditions were tested. As standard conditions a gas flow of 12 sccm was used for both oxygen and hydrogen together with a plasma power of 200 W. It can be noted that increasing the hydrogen plasma treatment time is important for increasing the SERS intensity. Using a hydrogen plasma time smaller than the oxygen plasma treatment time leads to incomplete reduction of the oxidized silver film and a lower SERS intensity. The use of equal treatment times using oxygen and hydrogen plasma (20 min O_2_ plasma and 20 min H_2_ plasma) increases the SERS intensity further. Using a hydrogen plasma treatment time is higher than that used for oxygen plasma results in a higher SERS enhancement, as it is observed for 15 min oxygen plasma and 20 min hydrogen plasma treatment due to the more complete reduction of the oxidized silver films. Increasing the hydrogen plasma reduction treatment time to 30 min does not lead to a further increase in the SERS enhancement. Instead there is a slight decrease in the SERS enhancement as excess hydrogen plasma treatment beyond the reduction process results in etching of the silver film: This widens the pores of the formed nanoporous silver network and decreases the SERS enhancement. A decrease in the used gas flow for oxygen and hydrogen as well as the decrease of the used plasma power will result in a decrease in the SERS enhancement with the latter having a more pronounced effect on the SERS enhancement. At low plasma power the surface is modified to a much lower extent leading to a much less nanoporous network (Figure S13, Supporting Information File 1), which is the reason for the low SERS intensities obtained.

To understand if the plasma gas itself used for the reduction process has an effect on the SERS performance, reduction with (i) argon and (ii) nitrogen was studied using oxygen as well as air as oxidizing plasma (Figure 14c,d). The obtained SERS intensities for 10^−6^ M RhB at 621 cm^−1^ (excitation wavelength 632.8 nm) reveal that argon plasma is more efficient irrespective of the oxidizing plasma used. This can be attributed to the lower reduction efficiency of nitrogen plasma [86].

#### Comparison of the enhancement performance of different SERS substrates

**Figure 15:**
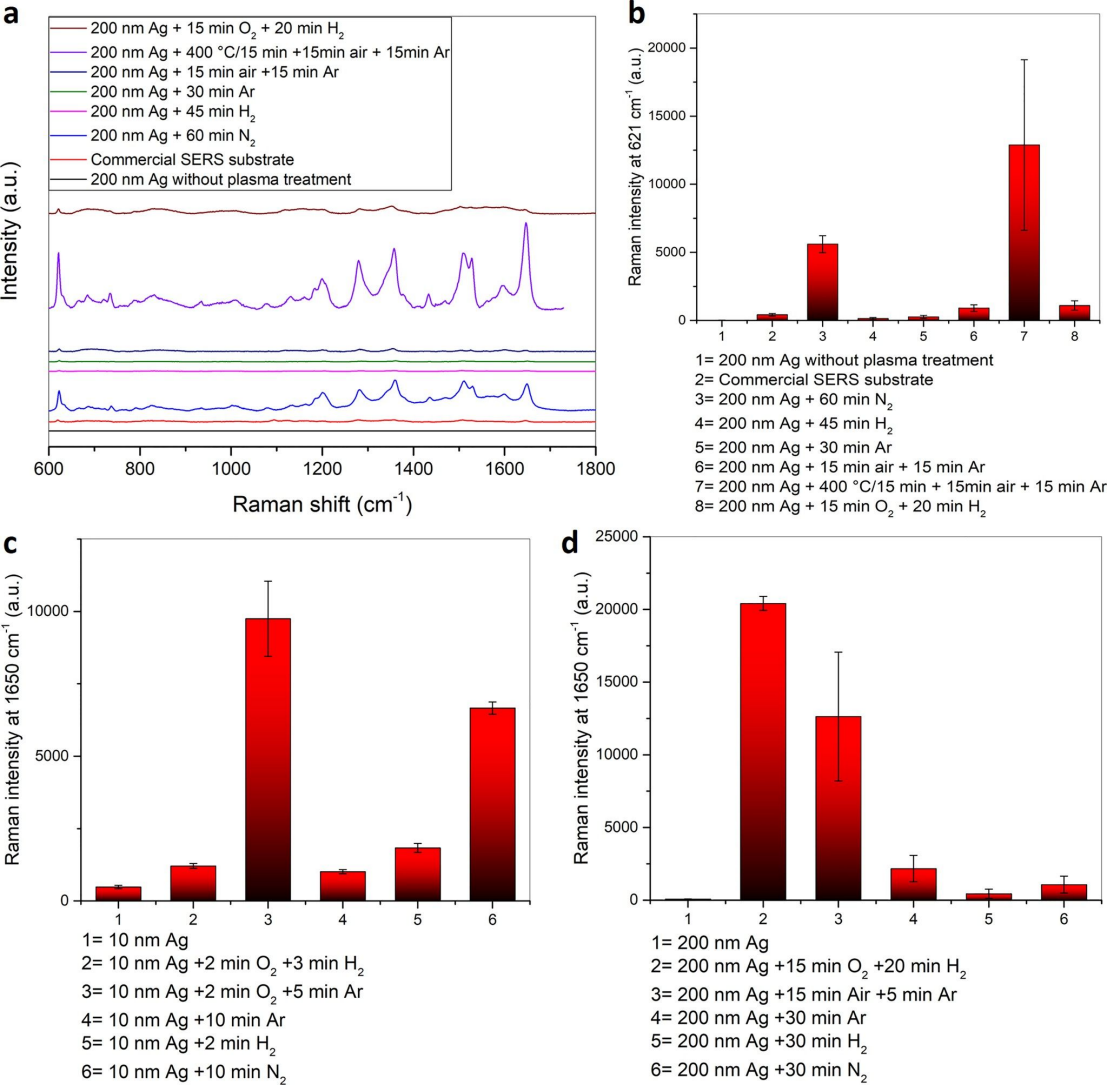
(a) Comparison of the SERS spectra of 10^−6^ M RhB on different SERS substrates and on a commercial SERS substrate using a 632.8 nm laser. (b) Comparison of the average Raman intensities at 621 cm^−1^. Comparison of the average Raman intensities at 1650 cm^−1^ on different SERS substrates produced through different plasma treatments on (c) 10 nm and (d) 200 nm sputtered silver films using a 532 nm laser.

A comparison of different SERS substrates a) a 200 nm sputtered silver film modified by different plasma treatments, b) an as-sputtered 200 nm silver film and c) a commercial SERS substrate (SEM, Figure S19, Supporting Information File 1) consisting of silver nanoparticles on a paper substrate using 10^−6^ M RhB as probe at an excitation wavelength of 632.8 nm is given in Figure 15a,b and Table S3 (Supporting Information File 1). As expected the commercial SERS substrate displays a higher SERS activity than the as-sputtered 200 nm silver film. However, the SERS enhancement of the as-sputtered 200 nm silver film can be greatly enhanced by an appropriate plasma treatment leading to a 30-fold enhancement compared to a commercial SERS substrate and to an about 1200-fold enhancement when compared with an untreated 200 nm sputtered silver film.

A further comparison of the SERS substrates was done at an excitation wavelength of 532 nm (Figure 15c,d and Table S3, Supporting Information File 1). A comparison of the group of the plasma treated ultrathin 10 nm silver films and the plasma treated 200 nm silver films shows that the SERS intensity obtained with the untreated 10 nm silver film is higher than with the untreated 200 nm film (Figure 15c,d and Table S3). For both thicknesses a plasma treatment results in an increase in the SERS intensity. However, in case of 10 nm silver films, a short plasma treatment of up to 10 min is already sufficient to yield a significant increase in the SERS intensity of the analyte compared to the untreated silver films considering the single plasma treatments (argon, nitrogen or hydrogen). For these single plasma treatments, the ultrathin transparent silver films are superior compared to their thicker, non-transparent analogues. In case of the oxidation/reduction plasma treatment, the 200 nm silver films result in better SERS enhancements as they provide more volume for the formation of a continuous and deep nanoporous silver network. In the ultrathin 10 nm silver film the oxidation/reduction plasma treatment results in the formation of a number of isolated islands (Figure S20, Supporting Information File 1) compared to the continuous silver network as in case of the thicker 200 nm silver film. Therefore, in the 200 nm silver films more adsorption sites for the analyte are created and an increased SERS effect is observed for a particular concentration of RhB. Thus, for the 10 nm silver films hydrogen and argon were tested as reducing plasma. It was found that using argon plasma as the reducing plasma yielded SERS substrates with higher SERS enhancement probably due to the fact that hydrogen plasma treatment partially results in etching of the silver islands in addition to the reduction. For the 200 nm silver films the oxidation/reduction plasma treatment is more efficient than a single plasma treatment in the formation of efficient SERS substrates.

**Figure 16:**
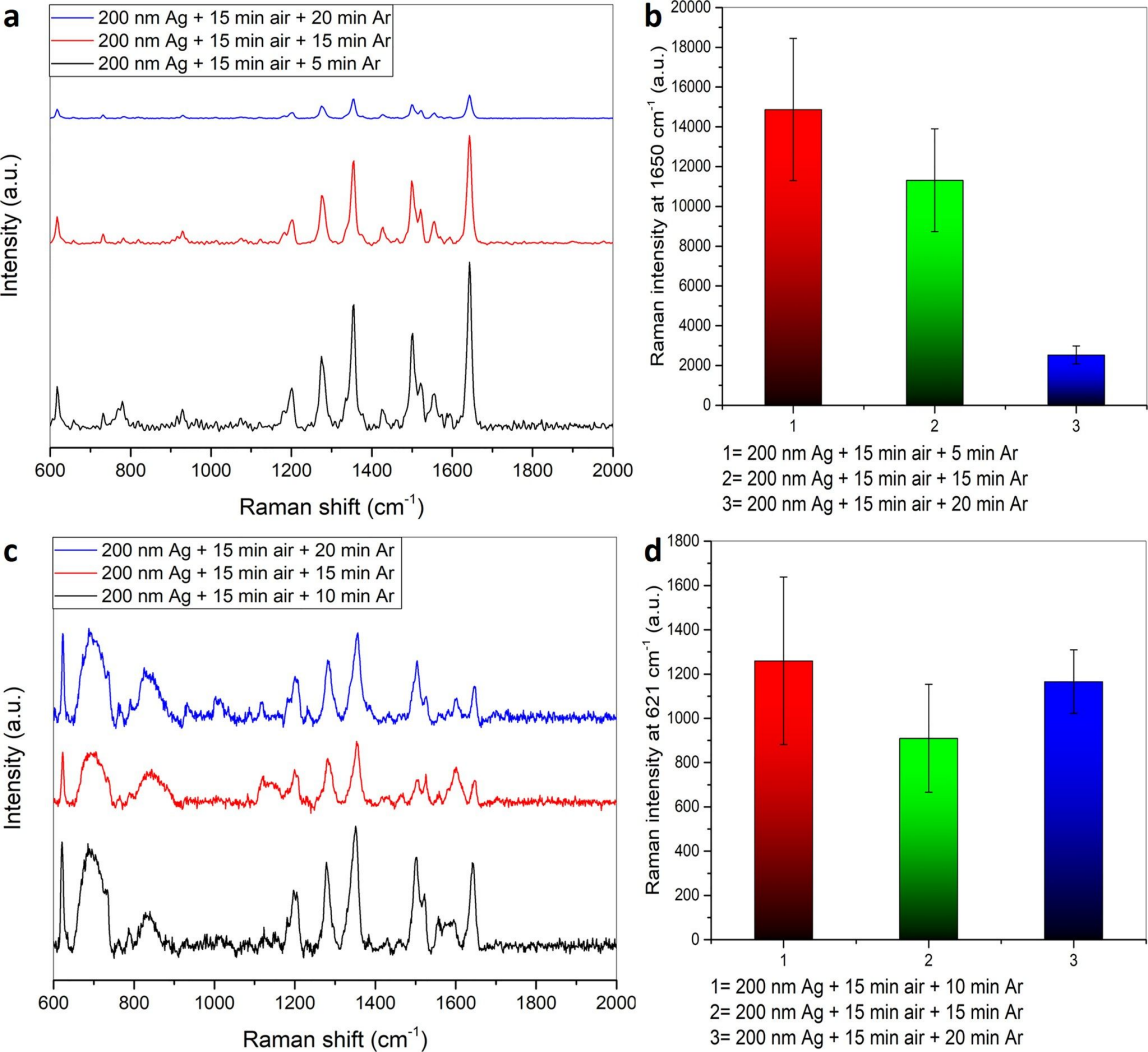
Comparison of the SERS spectra of 10^-6^ M RhB on different SERS substrates prepared through oxidation of 200 nm silver film with air plasma (g12-p200) for 15 min followed by reduction with an argon plasma (g16.7-p200) for different times using (a) 532 nm and (c) 632.8 nm laser. Comparison of the average Raman intensities at (b) 1650 cm^-1^ (532 nm laser excitation) and (d) 621 cm^-1^ (632.8 nm laser excitation) on the corresponding substrates.

In order to scrutinize the wavelength-dependence of different SERS substrates a comparison of SERS substrates produced by oxidation through air plasma and reduction through argon plasma with different reduction times was undertaken (Figure 16). The SERS performance of these substrates subjected to lower argon plasma reduction time at 532 nm excitation is much higher and the performance decreases rapidly with increasing argon plasma reduction time. This trend cannot be seen when using 632.8 nm as excitation wavelength where the SERS performance does not change much with increasing argon plasma reduction time.

Raman mapping of the strongest vibration mode of RhB at ν = 1650 cm^−1^ (–COOH) on different Ag substrates (different thicknesses and plasma treatment conditions) reveals the overall uniformity of this characteristic absorption. Moreover, the different SERS substrates show good enhancement factors and uniformity as obtained from the Raman maps of 10^−6^ M concentration of RhB on these substrates (Figure S21, Supporting Information File 1).

It is known that the SERS activity can deteriorate due to surface oxidation of the silver surface [88]. For increasing the long-term stability of SERS active silver films we have studied a thin layer of dielectric Al_2_O_3_ as protective coating [89]. A 1 nm thick Al_2_O_3_ film was deposited on top of the nanoporous silver film by ALD before adding the probe molecule RhB. The influence in SERS activity was compared immediately after deposition and after two weeks. Not unexpectedly, the Al_2_O_3_ layer reduces the SERS signal significantly as the analyte molecules are not in direct contact with the silver surface anymore (Figure S22, Supporting Information File 1). However, characteristic signals of RhB are still detectable and allow for a secure identification of RhB. Even after two weeks of storage the SERS substrate without Al_2_O_3_ exhibits great SERS performance superior to that of the Al_2_O_3_-coated SERS substrate.

The idea of using plasma treatment for cleaning the used SERS substrates without greatly modifying their response was also tested. This would enable an inexpensive re-use of the SERS substrates. First, the SERS spectrum of 10^−6^ M RhB on a 200 nm sputtered silver film was obtained. An argon plasma with a gas flow of 16.7 sccm at 50 W for 5 min was sufficient to remove the analyte completely (Figure S23a, Supporting Information File 1). Re-application of RhB to the cleaned SERS substrate gave an SERS spectrum equivalent to that before cleaning which proofs that the surface has not been altered extensively by the mild plasma cleaning procedure. A second attempt to clean the SERS substrate under the same conditions also completely removes the analyte providing a clean baseline. The same procedure has been also tested on an older SERS substrate (200 nm Ag + 15 min O_2_ + 20 min H_2_) that was fabricated six months prior to its use (Figure S23b, Supporting Information File 1). Before the first use it was cleaned using the mentioned short cleaning procedure with argon plasma. Then the same substrate was re-used four times with the short cleaning procedure with argon in-between. No decrease of the performance of the SERS substrate was observed. This shows that mild and short plasma treatment can be used effectively for cleaning of the SERS substrates, whereas more aggressive and longer plasma treatment can be used to increase the silver surface roughness and complexity yielding more effective SERS substrates from simple silver films.

## Conclusion

This systematic study proves the possibility of increasing the surface roughness of sputtered silver films through application of different rf plasma treatments as an alternative to the widely employed method of electrochemical surface roughening of silver films. Different reactive plasma gas compositions as well as different plasma parameters used in the particular plasma treatments result in a variety of different SERS silver substrates with tunable morphology and attractive enhancement factors. A combination of an oxidizing plasma together with a reducing plasma results in the formation of complex three-dimensional silver morphologies showing a huge enhancement factor due to the formation of SERS hot spots. The SERS enhancement of the as-sputtered 200 nm silver film is greatly enhanced by an appropriate plasma treatment reaching about 30-fold enhancement compared to a commercial SERS substrate and about 1200-fold compared to the untreated silver film. At the same time it was also found that ultrathin transparent silver films can be used as efficient SERS substrates. Heating of the silver film under a subsequent oxidative/reductive rf plasma treatment enables the formation of a silver-nanosponge morphology directly on the glass substrate. The morphologies fabricated by various plasma treatment processes are stable over the course of several weeks and can even be recovered and re-used after initial analytical use through employing a mild argon plasma treatment.

To summarize, rf plasma treatment offers flexibility in obtaining a wide variety of silver morphologies on a substrate starting from a thin sputter deposited silver film. We believe that this technique offers an attractive and efficient way for a straightforward fabrication of SERS substrates even based on other host metals.

## Supporting Information

File 1 Additional experimental data.
