# Supplementary material for: Controlling surface morphology and sensitivity of granular and porous silver films for surface-enhanced Raman scattering, SERS
Source: Beilstein J Nanotechnol. 2018 Nov 7;9:2813–31. doi: 10.3762/bjnano.9.263 (PMC6244324; doi:10.3762/bjnano.9.263)
Supplement: File 1 — Additional experimental data. [file Beilstein_J_Nanotechnol-09-2813-s001.pdf]

Supporting Information

for

Controlling surface morphology and sensitivity of granular and porous silver films for surface-enhanced Raman scattering, SERS

Sherif Okeil and Jörg J. Schneider*

Address: Eduard-Zintl-Institut für Anorganische und Physikalische Chemie, Technische Universität Darmstadt, Alarich-Weiss-Straße 12, 64287 Darmstadt, Germany

Email: Jörg J. Schneider* - joerg.schneider@ac.chemie.tu-darmstadt.de

* Corresponding author

Additional experimental data

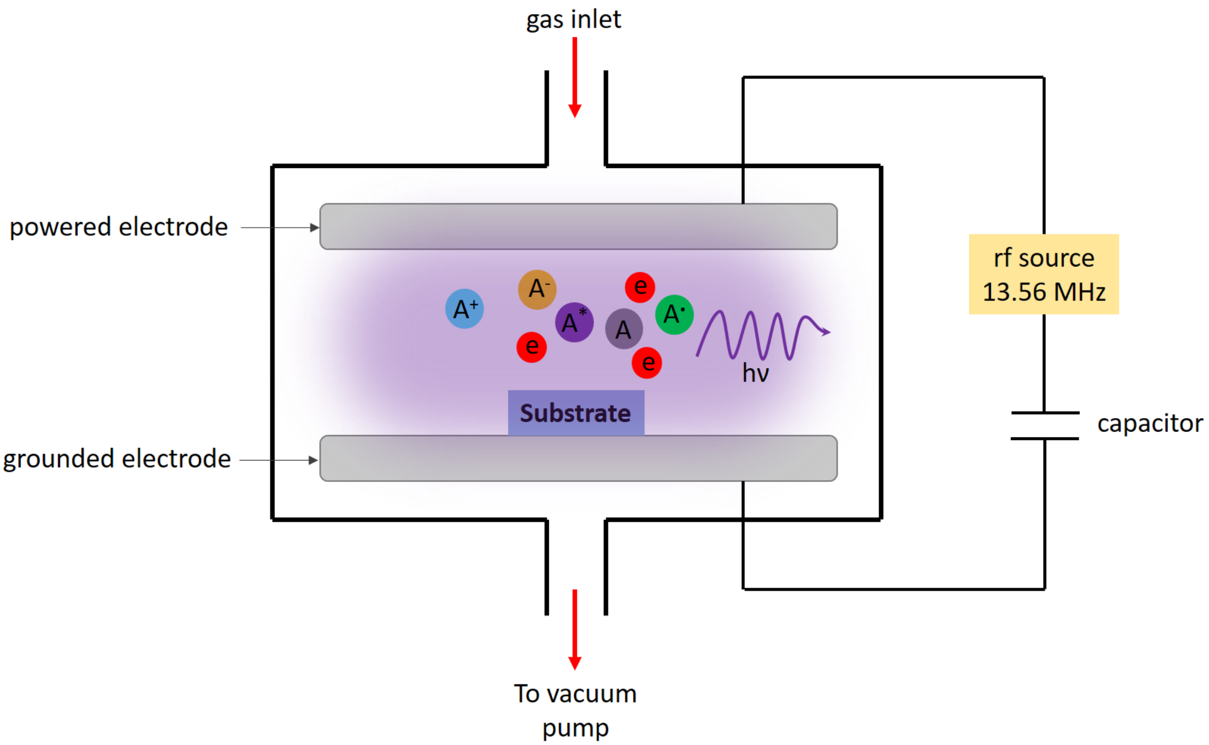

Scheme S1: Schematic representation of the parallel-plate capacitively coupled rf plasma.

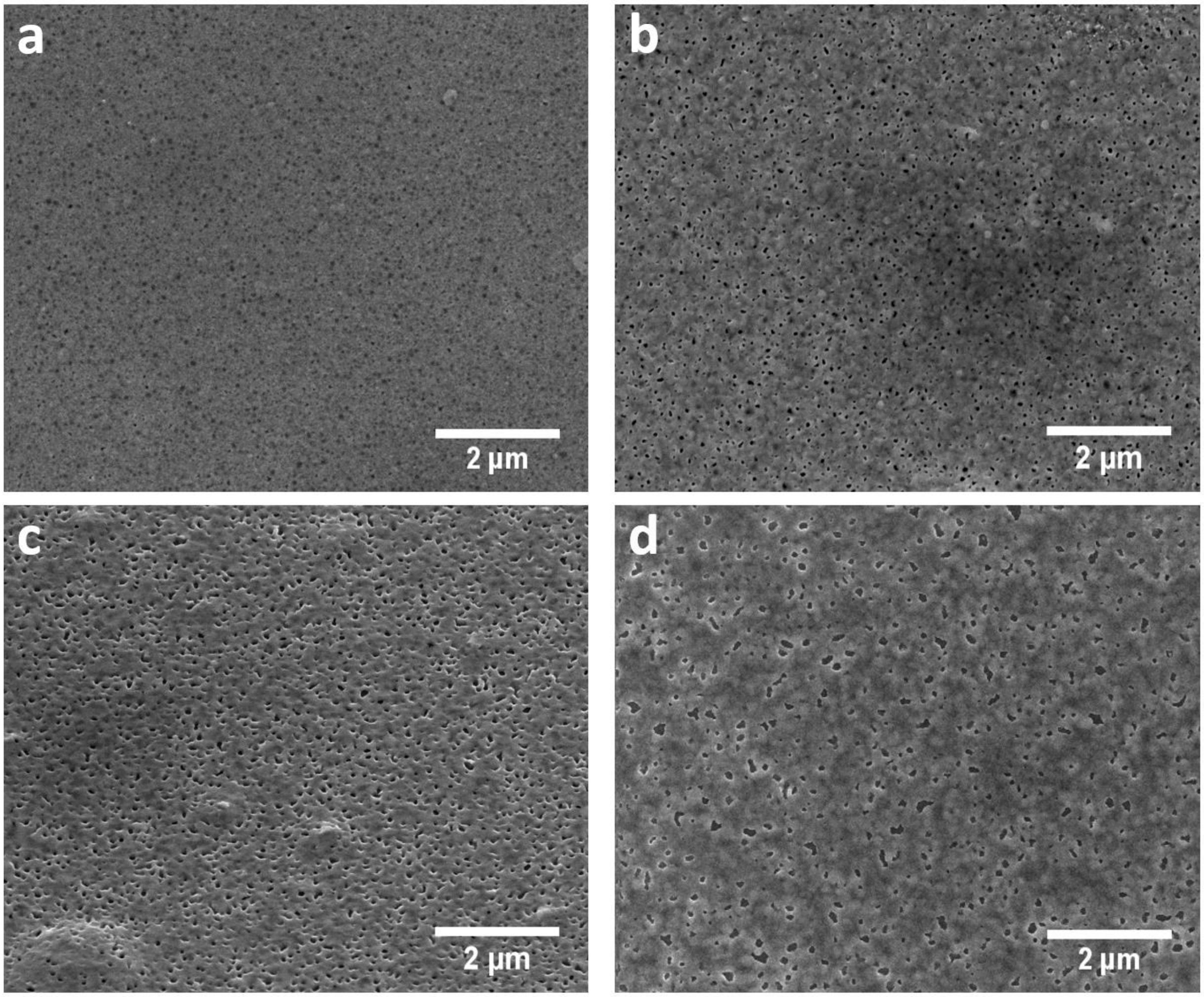

Figure S1: SEM image of 200 nm sputtered silver film treated with hydrogen plasma (g12-p200) for (a) 5 min, (b) 15 min, (c) 30 min (taken at 45° tilt) and (d) 45 min at 20000× magnification showing an overall view of the hydrogen plasma treated silver films.

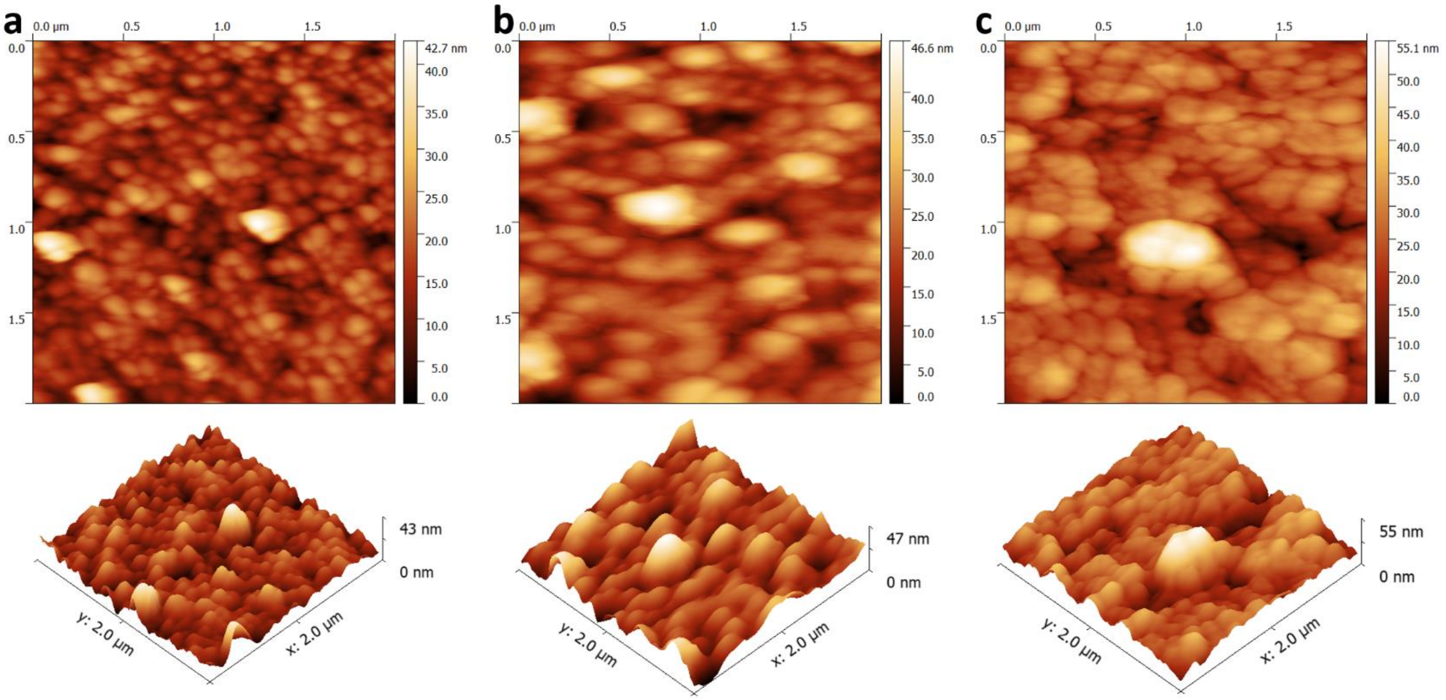

Figure S2: AFM topographical scans of 200 nm sputtered silver films treated with hydrogen plasma (g12-p200) for (a) 5 min (R_q = 5.03 nm), (b) 15 min (R_q = 6.29 nm) and (c) 45 min (R_q = 6.85 nm) revealing the increase in surface roughness with increasing hydrogen plasma treatment time.

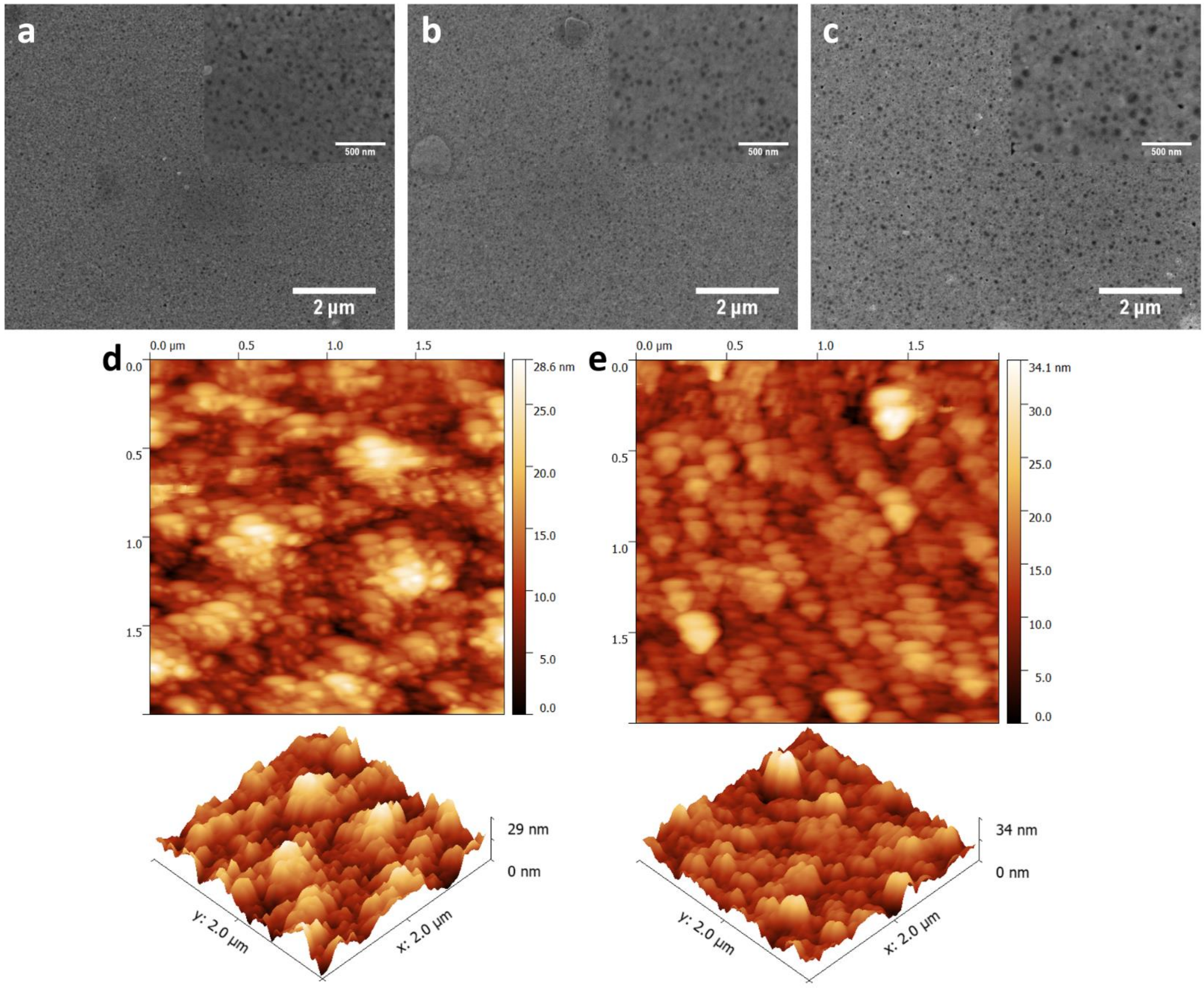

Figure S3: SEM image of 200 nm sputtered silver films treated with hydrogen plasma for 15 min with (a) g6-p50, (b) g12-p50 and (c) g12-p120 at 20000× magnification showing an overall view of the hydrogen plasma treated silver films. The inset shows the same SEM image at 100000x magnification. AFM topographical scans of 200 nm sputtered silver films treated with hydrogen plasma for 15 min with (d) g6-p50 (R_q = 4.41 nm) and (e) g12-p50 (R_q = 4.00 nm).

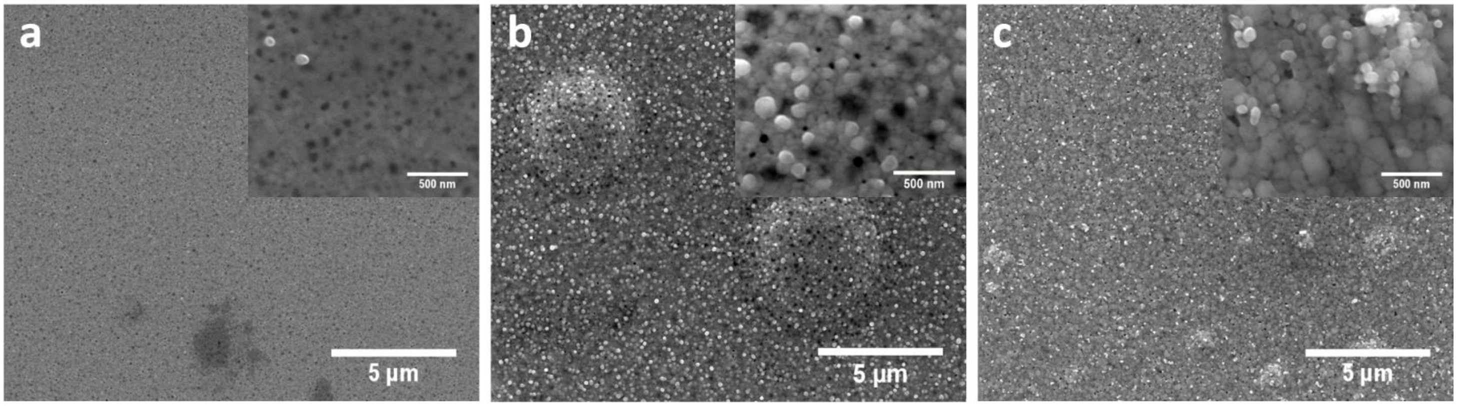

Figure S4: SEM image of 200 nm sputtered silver film treated with nitrogen plasma (g12-p200) for (a) 10 min, (b) 30 min and (c) 60 min at 10000× magnification showing an overall view of the nitrogen plasma treated silver films. The insets show the same SEM images at 100000× magnification.

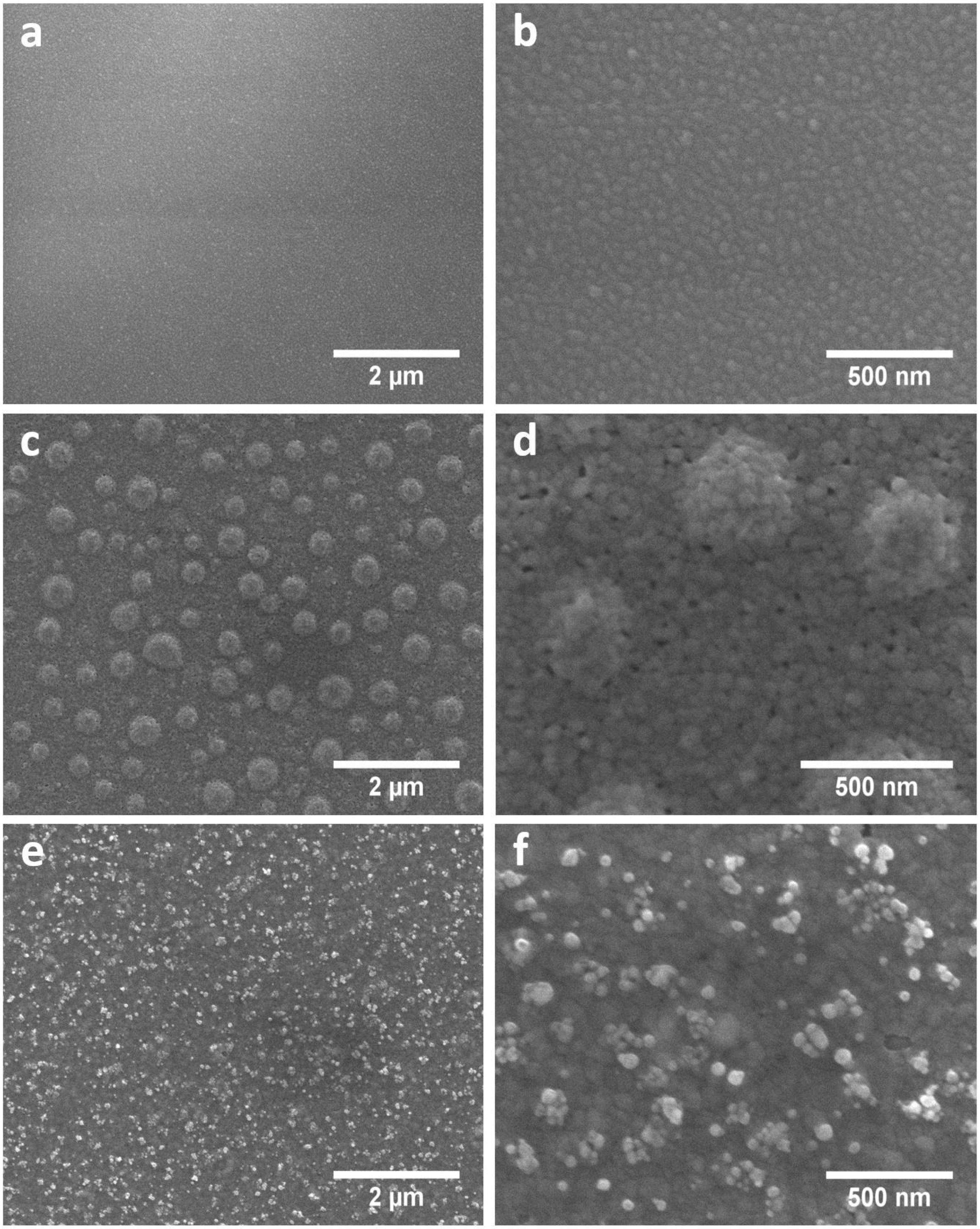

Figure S5: SEM image of (a) and (b) 10 nm sputtered silver film treated with nitrogen plasma (g12-p200) for 10 min at 25000× and 100000× magnification, respectively, (c) and (d) 50 nm sputtered silver film treated with nitrogen plasma (g12-p200) for 10 min at 25000× and 120000× magnification, respectively, (e) and (f) 50 nm sputtered silver film treated with nitrogen plasma (g12-p200) for 30 min at 25000× and 100000× magnification, respectively.

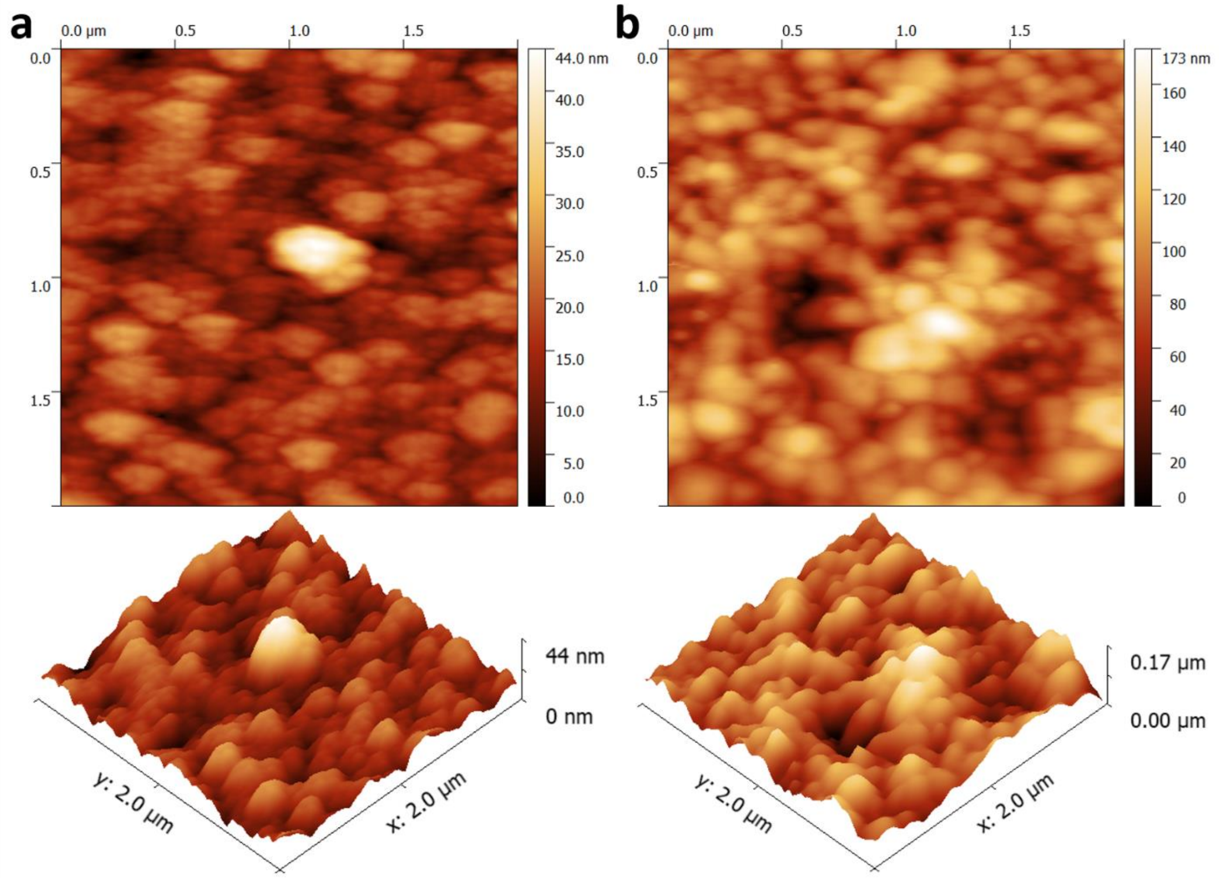

Figure S6: AFM topographical scans of 200 nm sputtered silver films treated with nitrogen plasma (g12-p200) for (a) 10 min (R_q = 5.12 nm) and (b) 60 min (R_q = 23.1 nm) showing the large increase in surface roughness with increasing nitrogen plasma treatment time.

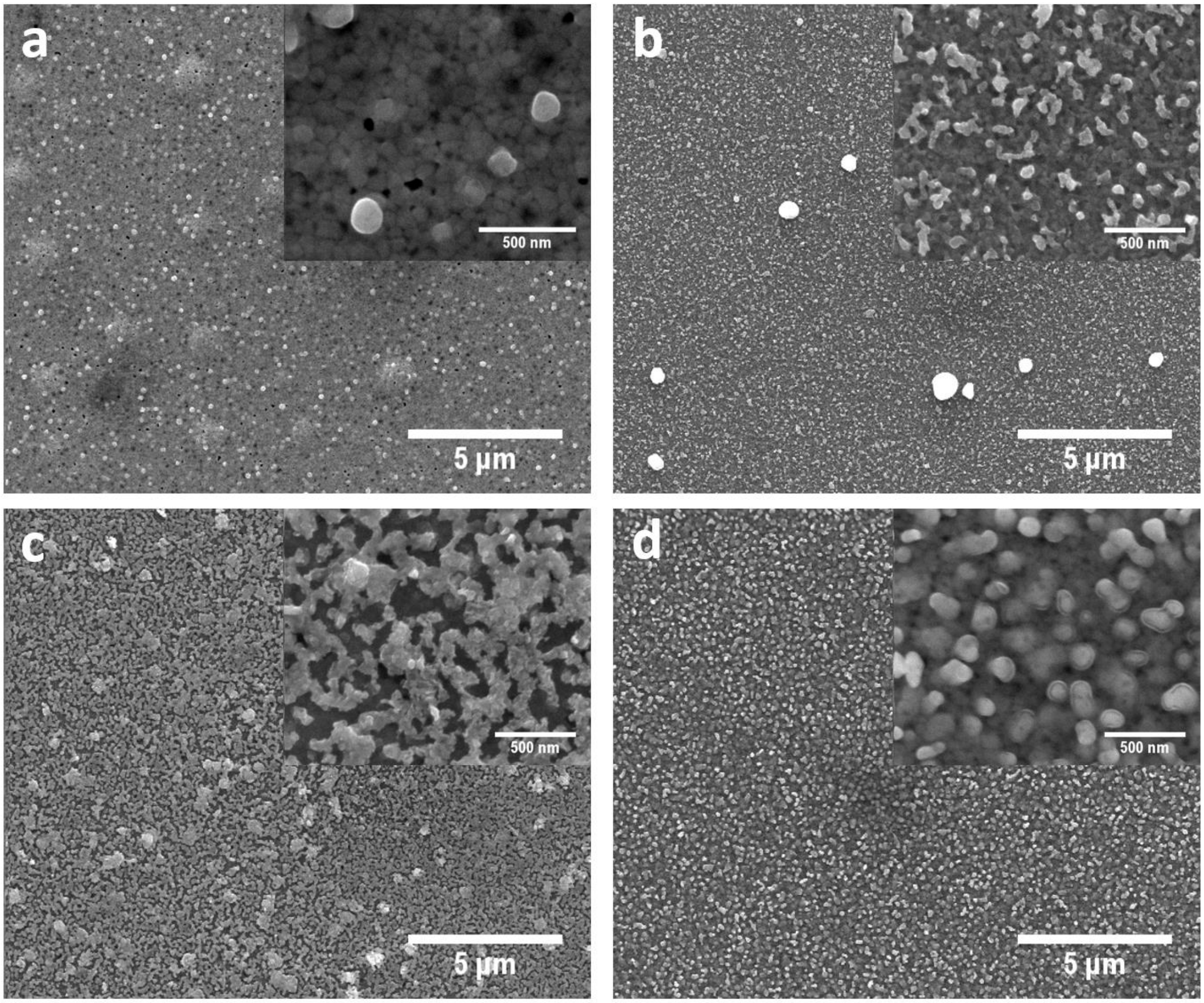

Figure S7: SEM image of 200 nm sputtered silver films treated with nitrogen plasma for 30 min with (a) g6-p200, (b) g6-p50, (c) g12-p50 and (d) g12-p120 at 10000× magnification showing an overall view of the nitrogen plasma treated silver films. The insets show the same SEM images at 100000× magnification.

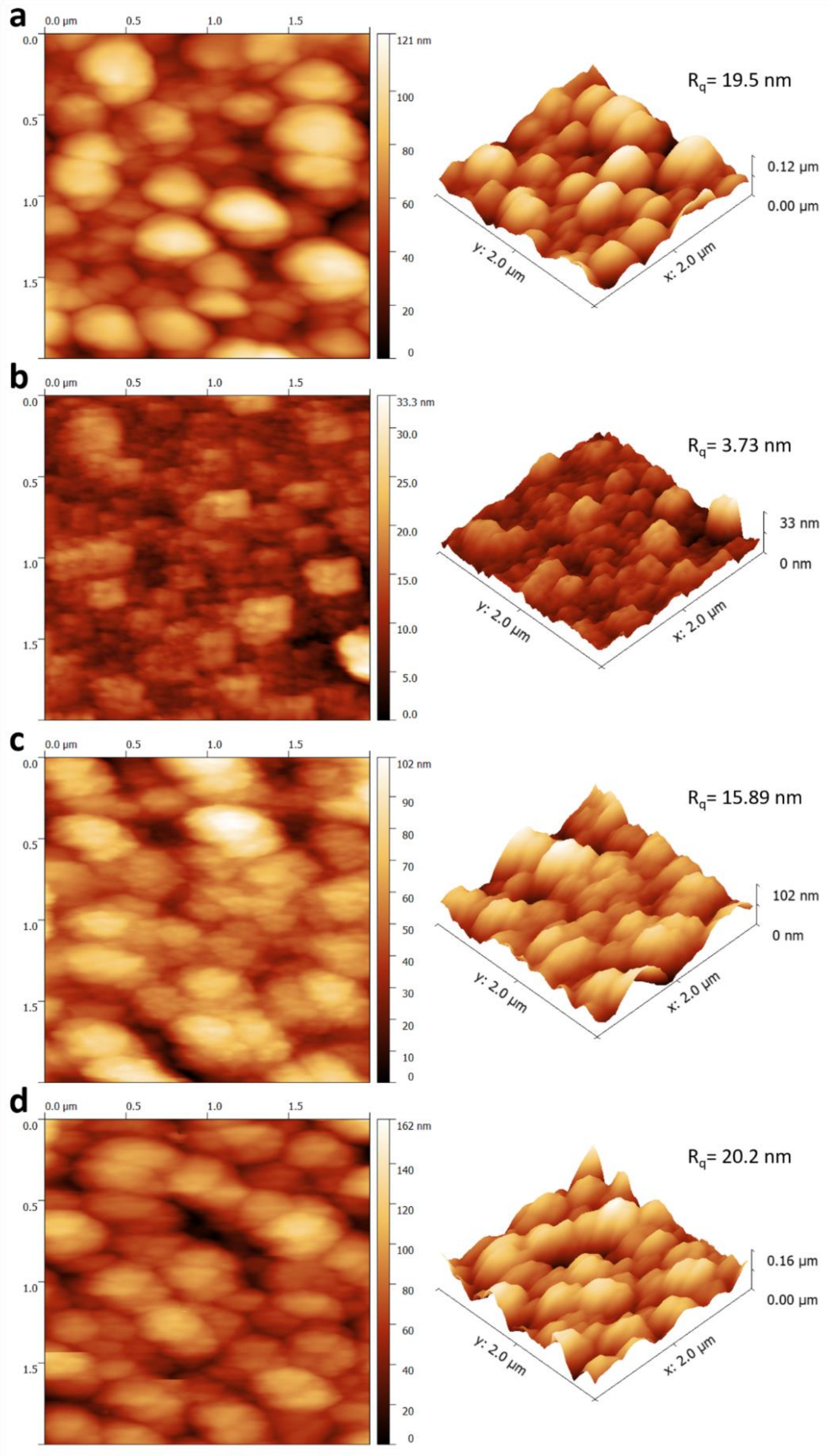

Figure S8: AFM topographical scans of 200 nm sputtered silver films treated with nitrogen plasma for 30 min with (a) g6-p200 (R_q = 19.5 nm), (b) g6-p50 (R_q = 3.73 nm), (c) g12-p50 (R_q = 15.89 nm) and (d) g12-p120 (R_q = 20.2 nm).

XPS and Auger spectroscopy analysis of as-sputtered silver films, hydrogen plasma treated silver films and nitrogen plasma treated silver films

XPS analysis of the different silver films shows the presence of carbon and oxygen. This is normal for silver films exposed to the atmosphere where adventitious carbon, chemisorbed oxygen as well as adsorbed carbonate species could be found on the silver surface [1,2].

The difference in the peak for adsorbed carbonate species when comparing as-sputtered silver with silver after plasma treatment, which can be also observed in the corresponding oxygen peak for adsorbed carbonate species, indicates that plasma treatment resulted in a significant change to the silver surface and could be due to the increase in surface area.

The presence of sodium in the plasma treated samples can be only explained by the migration of sodium from the soda–lime glass substrates during plasma treatment.

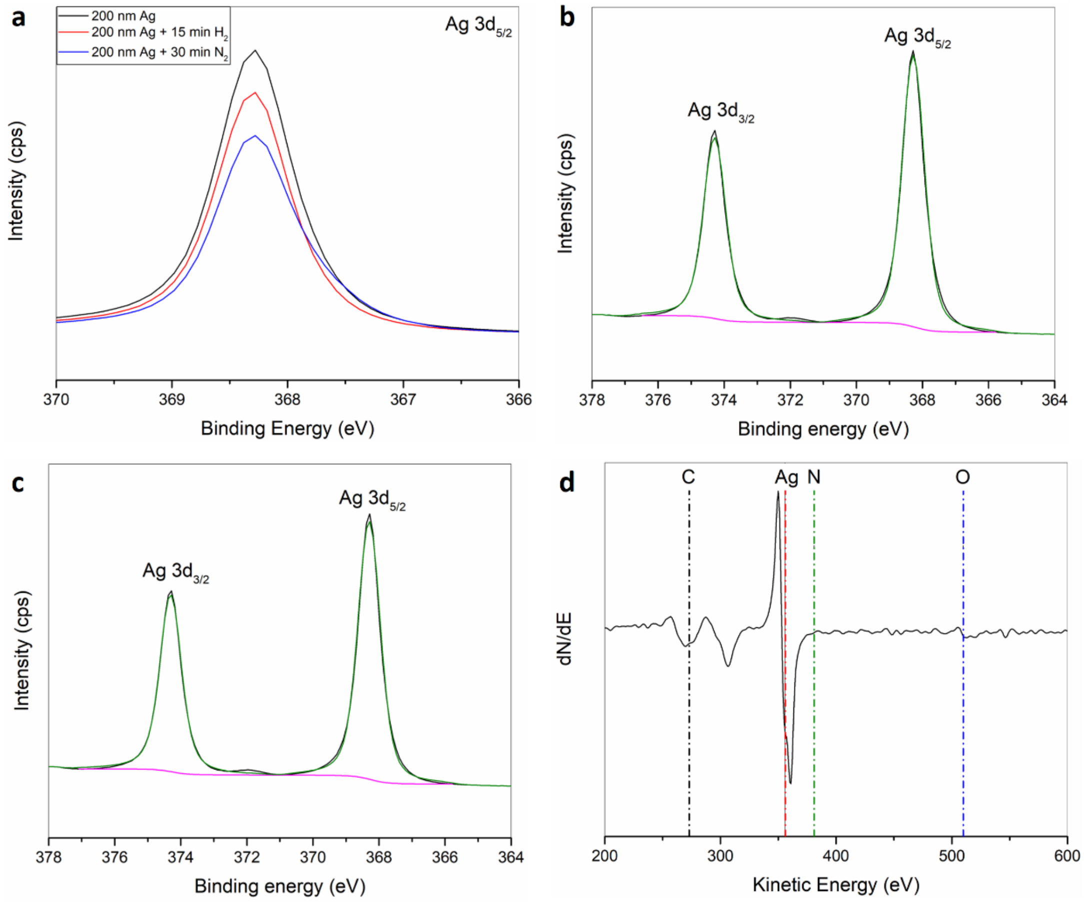

Figure S9: (a) XPS Ag $3d_{5/2}$ spectra recorded for 200 nm Ag, 200 nm Ag + 15 min hydrogen plasma treatment (g12-p200) and 200 nm Ag + 30 min nitrogen plasma treatment (g12-p200). (b) Deconvoluted XPS Ag 3d spectrum for 200 nm Ag. (c) Deconvoluted XPS Ag 3d spectrum for 200 nm Ag + 15 min hydrogen plasma treatment (g12-p200). (d) Auger spectrum of 200 nm Ag + 30 min nitrogen plasma treatment (g12-p200) while the lines show the reference positions of carbon, silver, nitrogen and oxygen.

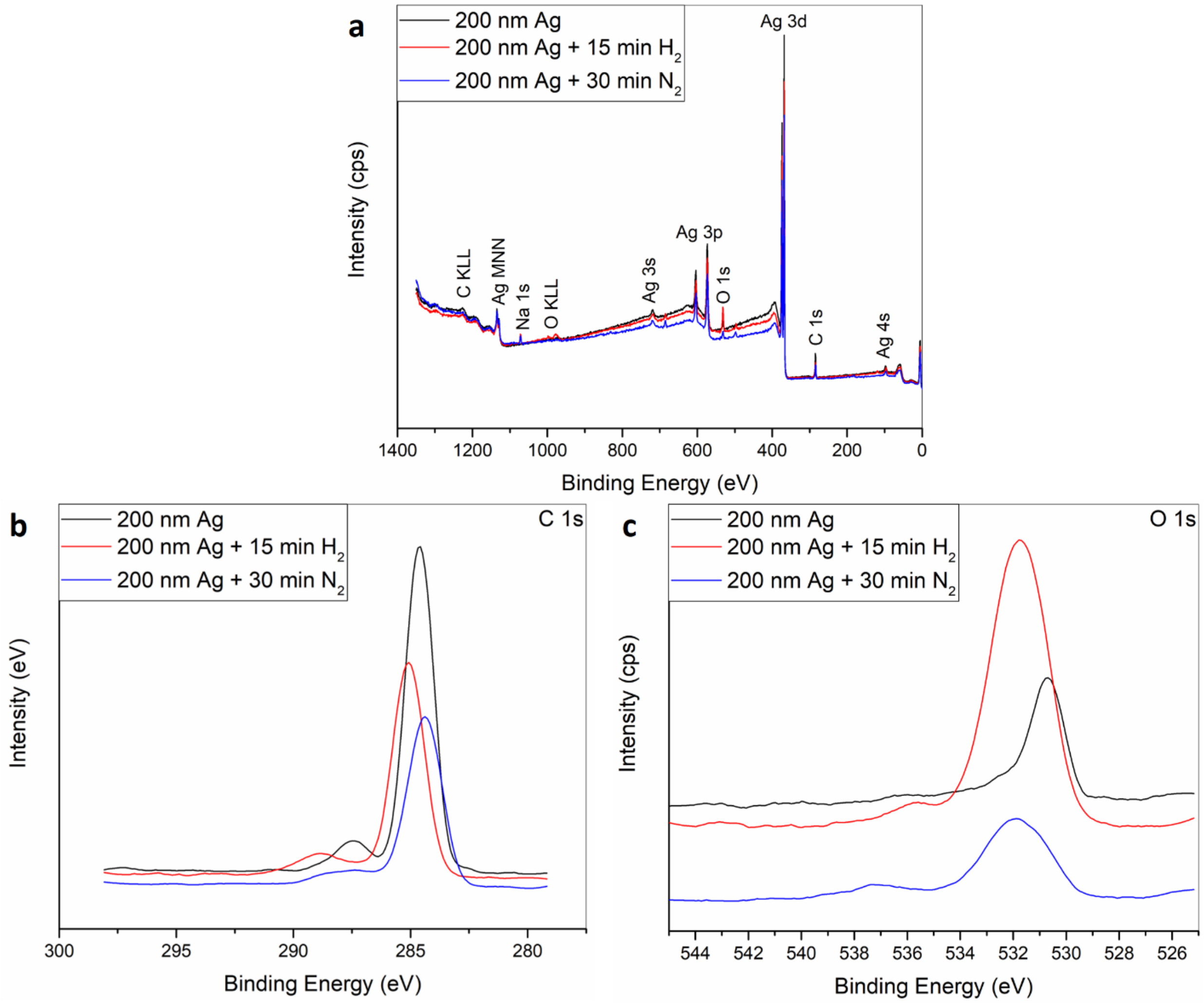

Figure S10: Comparison of the XPS (a) survey, (b) C 1s and (c) O 1s spectra of as-sputtered 200 nm Ag, 200 nm Ag + 15 min hydrogen plasma treatment (g12-p200) and 200 nm Ag + 30 min nitrogen plasma treatment (g12-p200).

Table S1: Atomic concentrations of the different elements detected by the XPS analysis of the different non-plasma treated and plasma treated silver films.

sample	concentration (atom %)			
	Ag	C	O	Na
as-sputtered 200 nm Ag	47.21	45.59	7.20	—
200 nm Ag + 15 min H_2 plasma (g12-p200)	38.53	33.95	23.53	3.99
200 nm Ag + 15 min N_2 plasma (g12-p200)	46.45	37.52	10.11	5.92

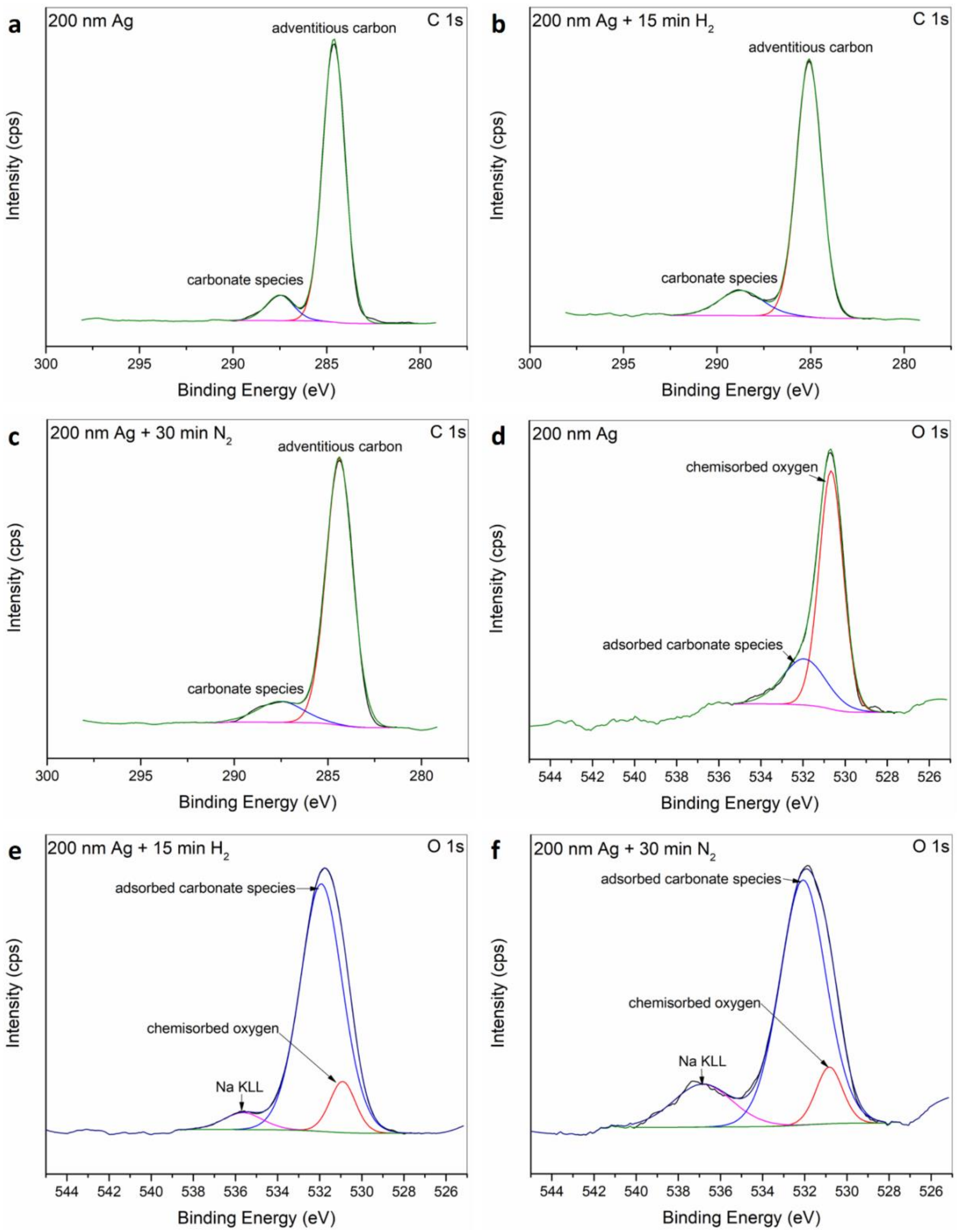

Figure S11: Deconvoluted XPS C 1s spectra of (a) as-sputtered 200 nm Ag, (b) 200 nm Ag + 15 min hydrogen plasma treatment (g12-p200) and (c) 200 nm Ag + 30 min nitrogen plasma treatment (g12-p200) showing adsorbed adventitious carbon and carbonate species. Deconvoluted XPS O 1s spectra of (d) as-sputtered 200 nm Ag, (e) 200 nm Ag + 15 min hydrogen plasma treatment (g12-p200) and (f) 200 nm Ag + 30 min nitrogen plasma treatment (g12-p200) showing the presence of adsorbed carbonate species and chemisorbed oxygen.

Table S2: XPS data for the deconvoluted C 1s and O 1s peaks of the different non-plasma treated and plasma treated silver films.

parameter	C 1s						O 1s					
	as-sputtered 200 nm Ag		200 nm Ag + 15 min H_2 plasma (g12-p200)		200 nm Ag + 15 min N_2 plasma (g12-p200)		as-sputtered 200 nm Ag		200 nm Ag + 15 min H_2 plasma (g12-p200)		200 nm Ag + 15 min N_2 plasma (g12-p200)	
binding energy (eV)	284.6	287.5	285.1	288.7	284.4	287.5	530.7	531.9	530.9	531.9	530.8	532.1
FWHM	1.37	1.72	1.56	2.74	1.65	2.87	1.33	2.43	1.42	2.35	1.48	2.57
% concentration relative to peak area	89.88	10.12	85.47	14.53	88.02	11.98	73.95	26.05	10.54	84.06	9.80	73.53

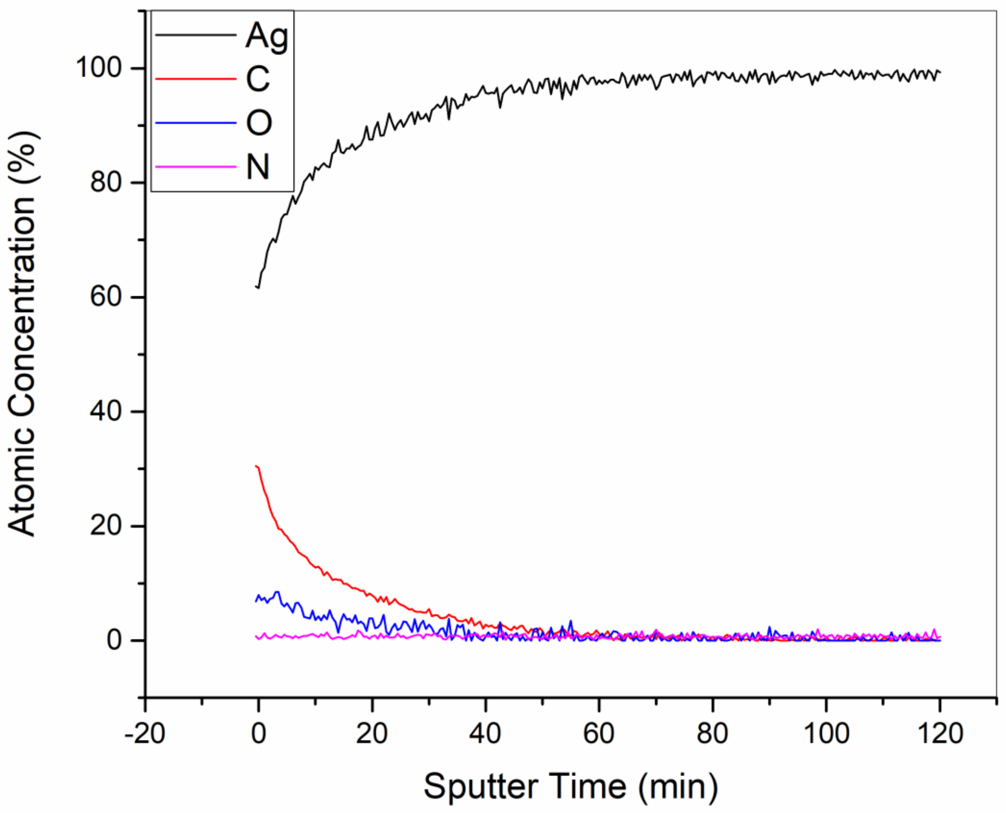

Figure S12: Auger depth profile for 200 nm Ag + 30 min nitrogen plasma treatment (g12-p200) showing the absence of nitrogen in the whole silver film.

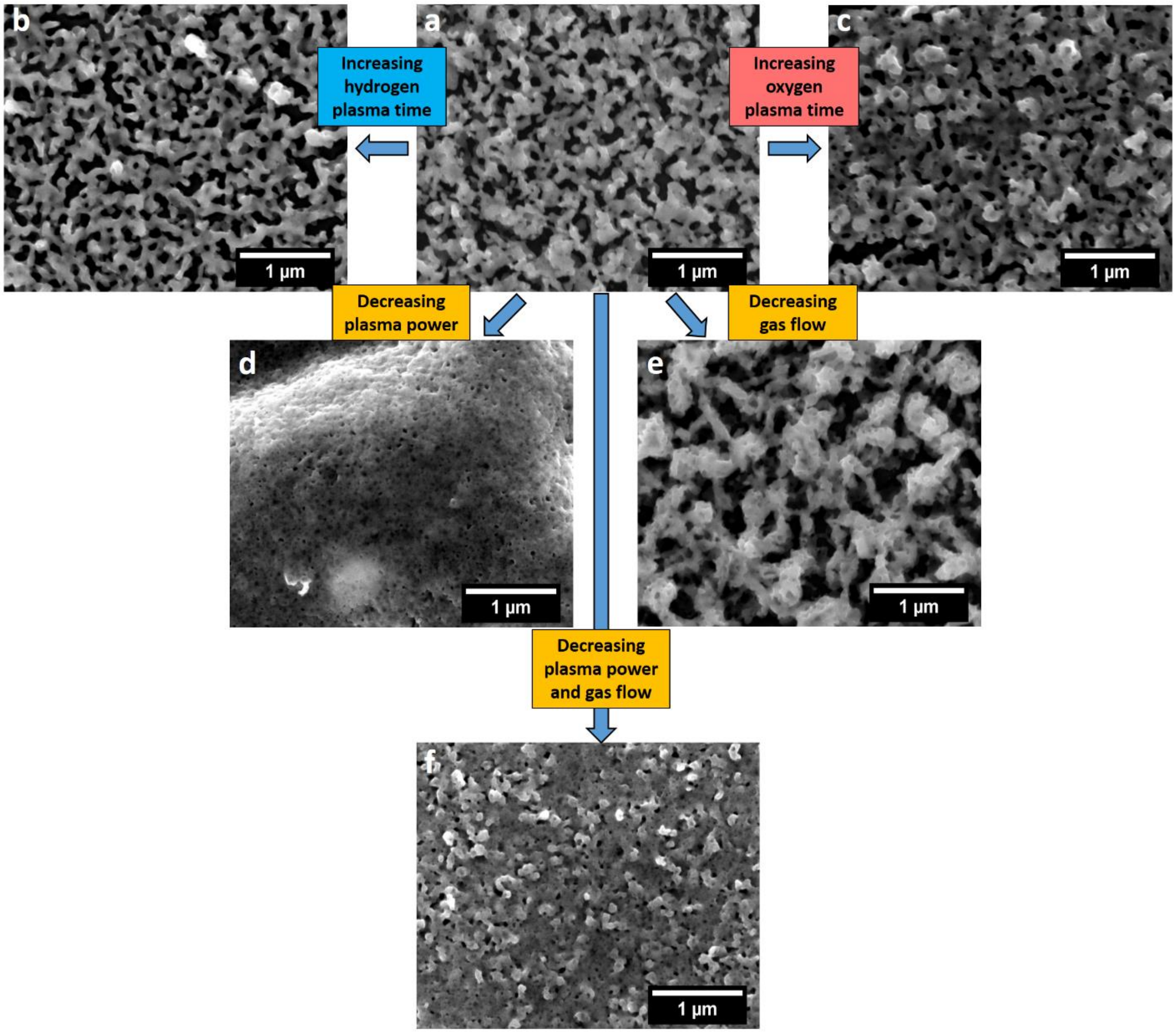

Figure S13: SEM image of 200 nm sputtered silver films treated with (a) oxygen plasma (g12-p200) for 15 min followed by reduction with hydrogen plasma (g12-p200) for 20 min, (b) oxygen plasma (g12-p200) for 15 min followed by reduction with hydrogen plasma (g12-p200) for 30 min, (c) oxygen plasma (g12-p200) for 20 min followed by reduction with hydrogen plasma (g12-p200) for 20 min, (d) oxygen plasma (g12-p50) for 15 min followed by reduction with hydrogen plasma (g12-p50) for 20 min, (e) oxygen plasma (g6-p200) for 15 min followed by reduction with hydrogen plasma (g6-p200) for 20 min and (f) oxygen plasma (g6-p50) for 15 min followed by reduction with hydrogen plasma (g6-p50) for 20 min.

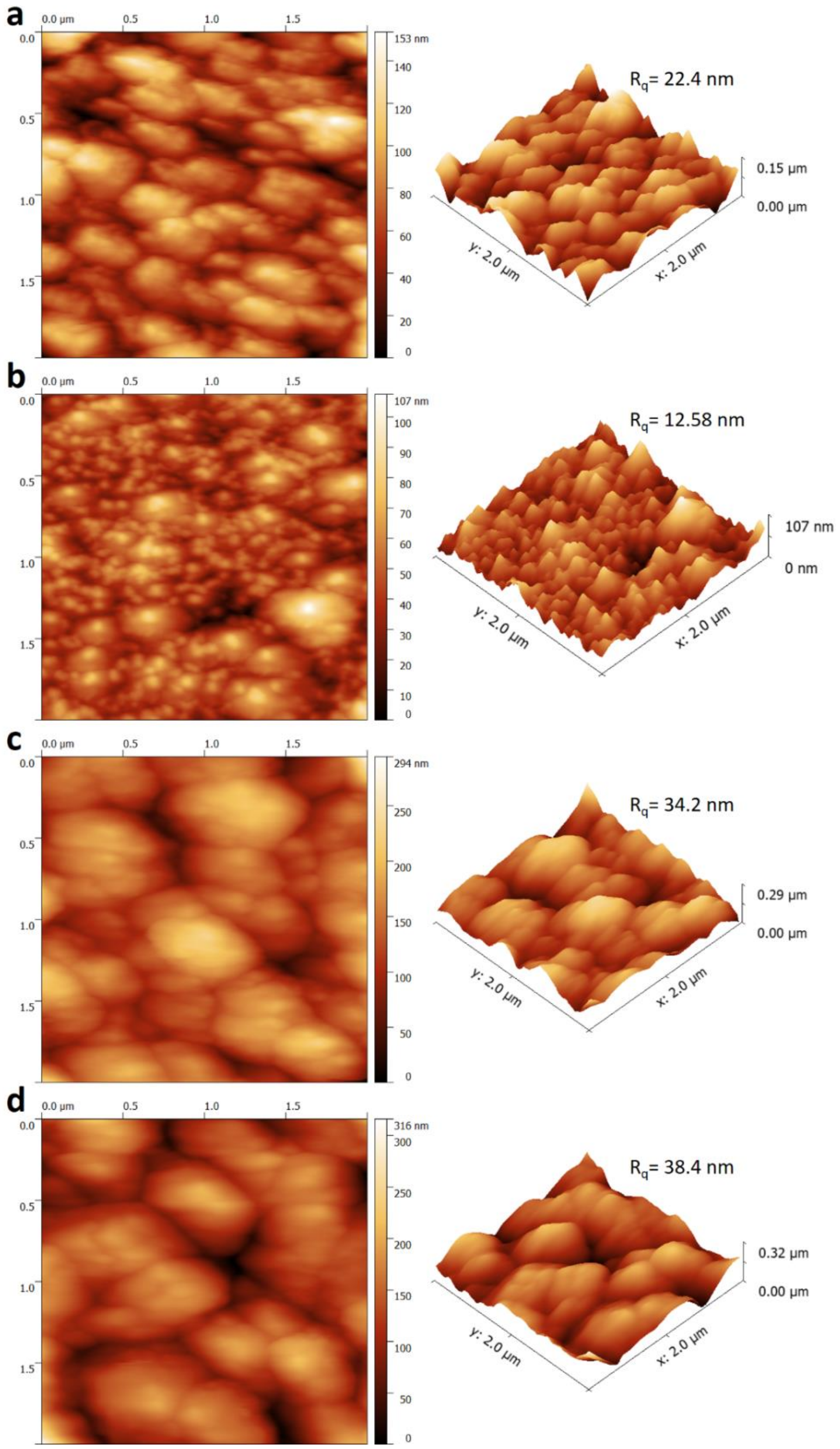

Figure S14: AFM topographical scans of 200 nm sputtered silver films treated with (a) 5 min oxygen and 7 min hydrogen plasma (g12-p200, R_q = 22.4 nm), (b) 15 min oxygen and 20 min hydrogen plasma (g12-p50, R_q = 12.58 nm), (c) 15 min oxygen and 20 min hydrogen plasma (g12-p200, R_q = 34.2 nm) and (d) 20 min oxygen and 20 min hydrogen plasma (g12-p200, R_q = 38.4 nm).

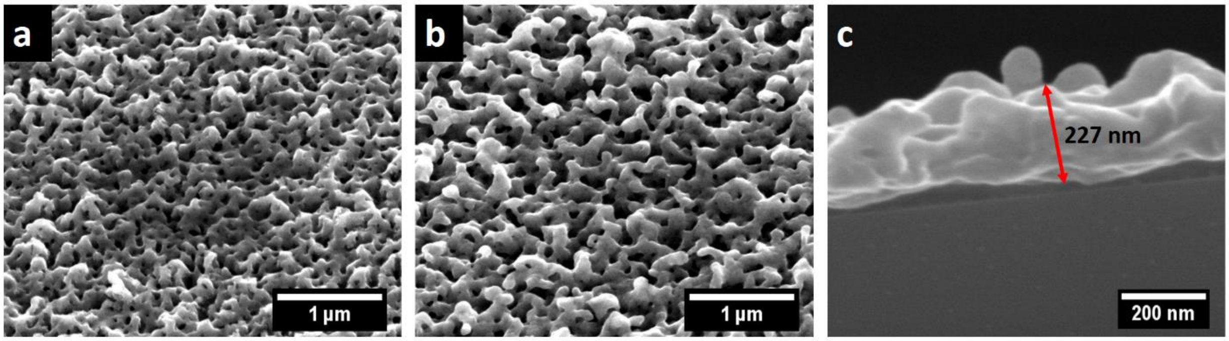

Figure S15: SEM image taken at 45° tilt of 200 nm sputtered silver film treated with (a) oxygen plasma (g12-p200) for 15 min followed by reduction with hydrogen plasma (g12-p200) for 20 min, (b) oxygen plasma (g12-p200) for 15 min followed by reduction with argon plasma (g16.7-p200) for 20 min. (c) Cross-sectional SEM for 200 nm sputtered silver film treated with oxygen plasma (g12-p200) for 15 min followed by reduction with argon plasma (g16.7-p200) for 20 min showing the actual thickness of the prepared silver film after plasma treatment.

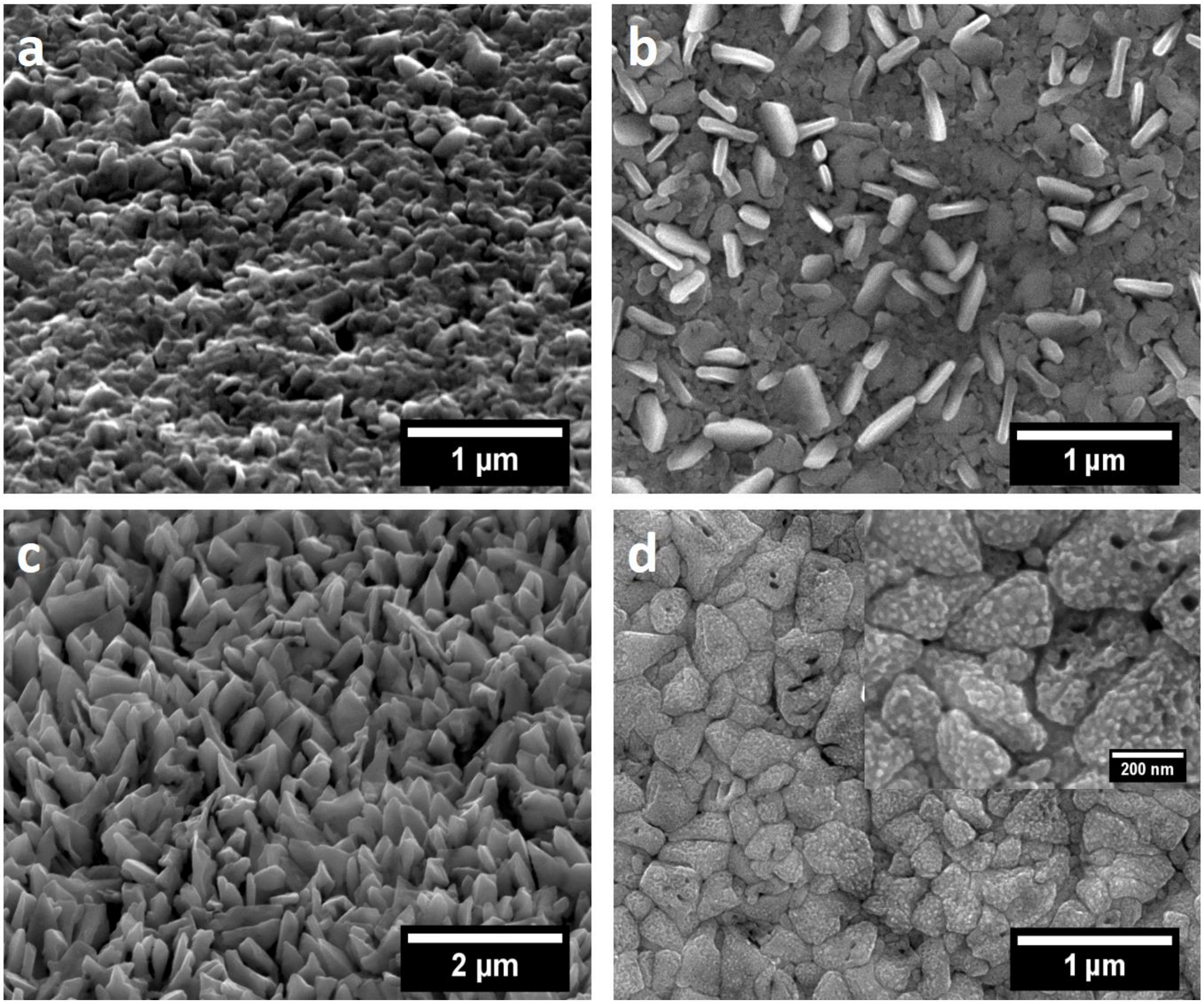

Figure S16: SEM image of 200 nm sputtered silver film oxidized with (a) oxygen plasma (g12-p200) for 15 min taken at 45° tilt, (b) air plasma (g12-p200) for 15 min, (c) air plasma (g12-p200) for 30 min taken at 45° tilt and (d) a mixture of argon (8 sccm) and oxygen (4 sccm) plasma (200 W) for 15 min; the inset shows the same SEM image at 200000× magnification.

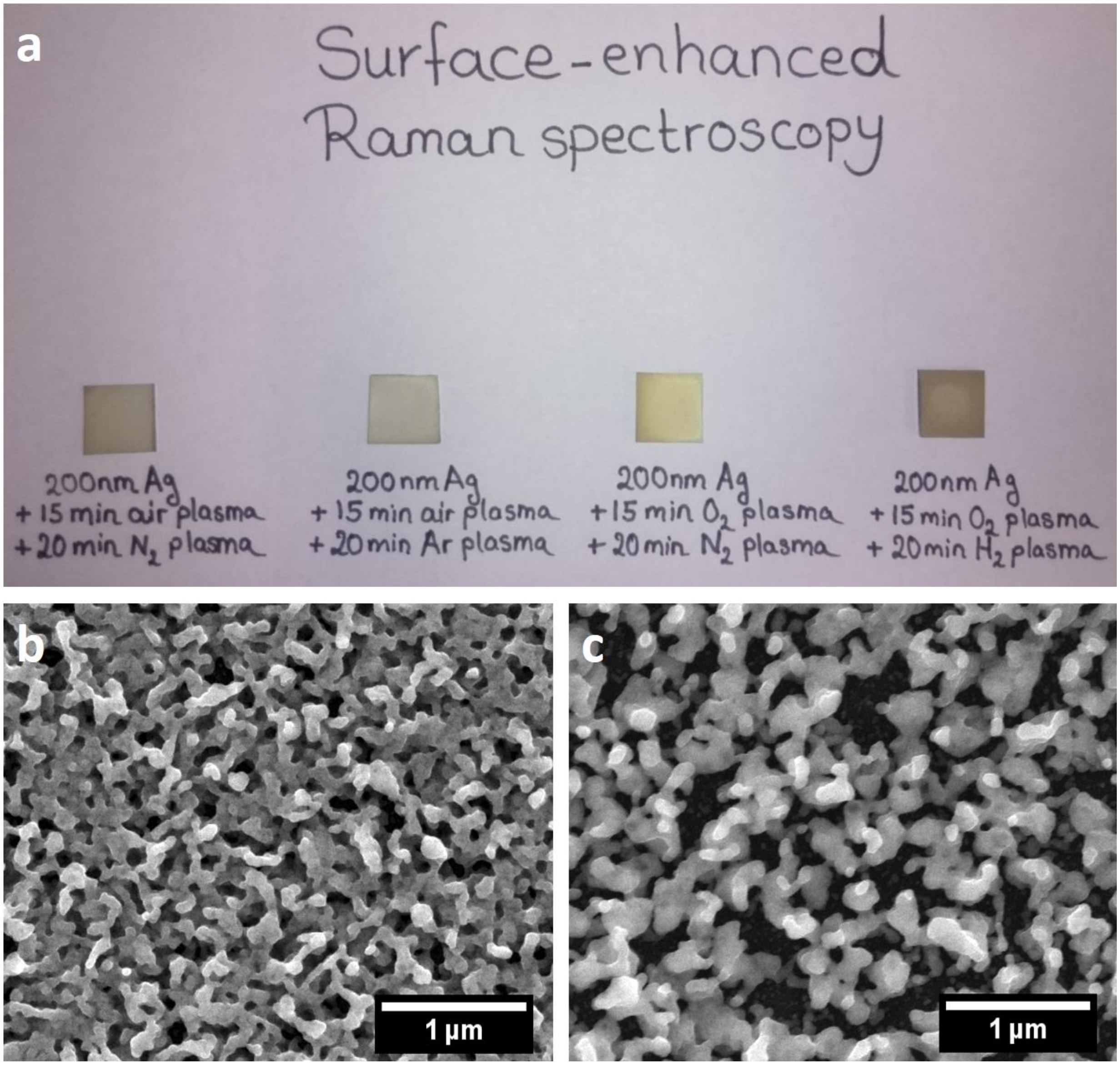

Figure S17: (a) Photographs of 200 nm silver films after different oxidation-reduction plasma treatments showing the difference in appearance resulting from different oxidation-reduction plasma treatments. SEM image of 200 nm sputtered silver film treated with (b) oxygen plasma (g12-p200) for 15 min followed by reduction with nitrogen plasma (g12-p200) for 20 min, (c) air plasma (g12-p200) for 15 min followed by reduction with nitrogen plasma (g12-p200) for 20 min.

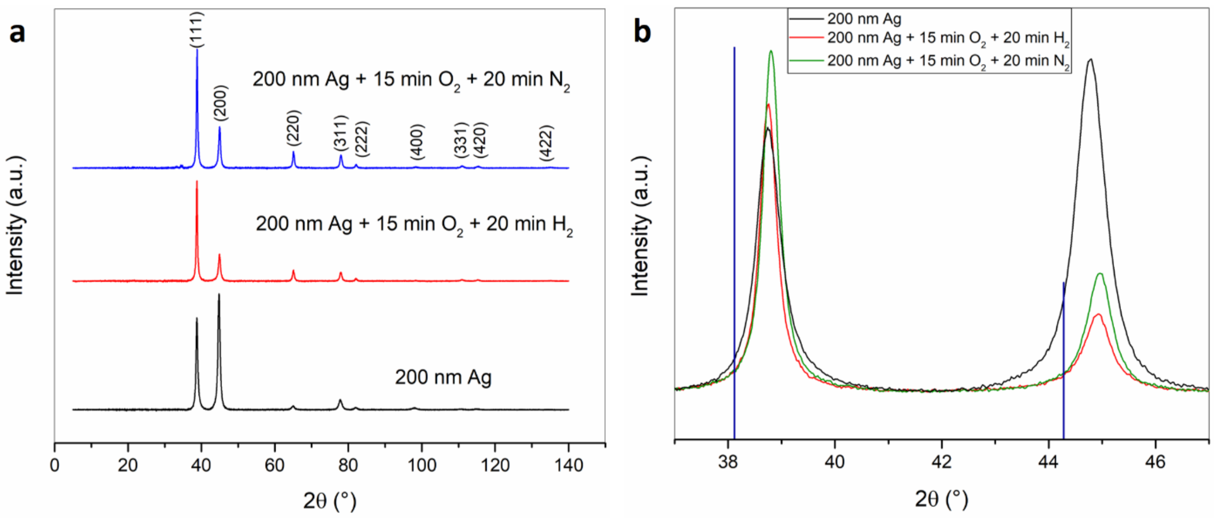

Figure S18: XRD of 200 nm Ag, 200 nm Ag + 15 min oxygen plasma (g12-p200) + 20 min hydrogen plasma treatment (g12-p200), 200 nm Ag + 15 min oxygen plasma (g12-p200) + 20 min nitrogen plasma treatment (g12-p200) and their comparison with standard XRD pattern for silver as reference (JCPDS file No. 04-0783).

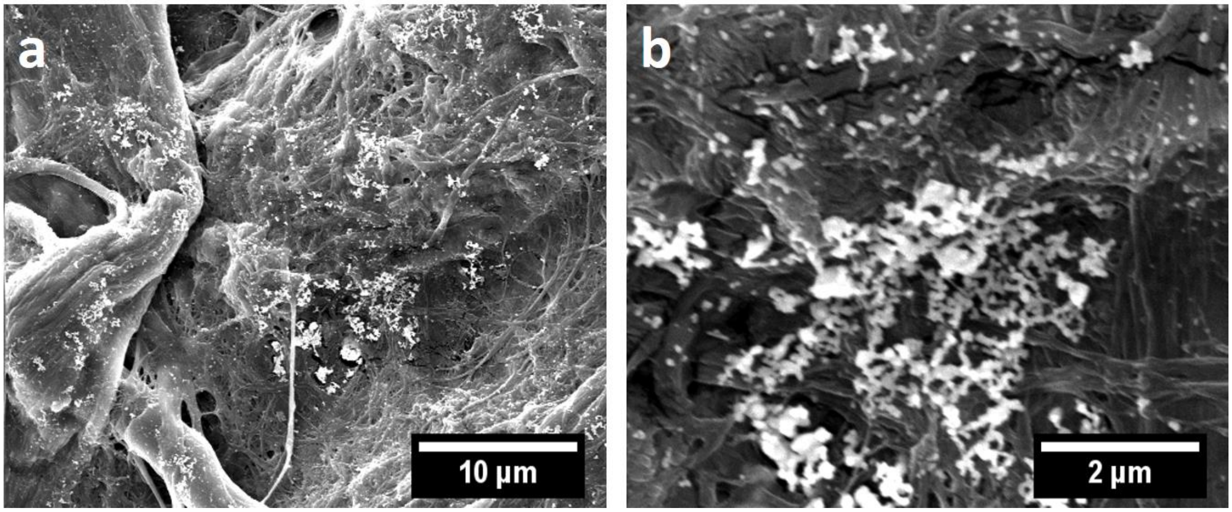

Figure S19: SEM image of a commercial SERS substrate at (a) 5000× and (b) 25000× magnification showing silver nanoparticles on a paper substrate.

Calculation of enhancement factors of different SERS substrates

Enhancement factors were calculated according to the equation

$$\mathrm{EF} = \frac{I_{\mathrm{SERS}}/C_{\mathrm{SERS}}}{I_{\mathrm{RS}}/C_{\mathrm{RS}}}$$

where C_{RS} and I_{RS} are the concentration (1×10^{-3} M) and Raman intensity for the regular Raman measurement of RhB on a glass substrate, respectively, and C_{SERS} and I_{SERS} are the concentration (1×10^{-6} M) and SERS intensity for the SERS measurement on the SERS substrate under investigation, respectively [3]. For an excitation wavelength of 532 nm the peak at 1650 cm^{-1} was used and for an excitation wavelength of 632.8 nm the peak at 621 cm^{-1} was used for the calculation of the enhancement factor as in each case this was the most intense peak in the obtained Raman spectrum.

Table S3: Enhancement factors obtained for different SERS substrates using 532 and 632.8 nm excitation wavelength.

	**enhancement factor**
**532 nm**	
10 nm Ag	5.55×10^{3}
10 nm Ag + 2 min O_2 +3 min H_2	1.39×10^{4}
10 nm Ag + 2 min O_2 + 5 min Ar	1.12×10^{5}
10 nm Ag + 10 min Ar	1.17×10^{4}
10 nm Ag + 2 min H_2	2.11×10^{4}
10 nm Ag +10 min N_2	7.66×10^{4}
200 nm Ag	7.87×10^{2}
200 nm Ag + 15 min O_2 + 20 min H_2	2.35×10^{5}
200 nm Ag + 15 min air + 5 min Ar	1.45×10^{5}
200 nm Ag + 30 min Ar	2.50×10^{4}
200 nm Ag + 30 min H_2	5.05×10^{3}
200 nm Ag + 30 min N_2	1.23×10^{4}
**632.8 nm**	
200 nm Ag	1.93×10^{2}
commercial SERS substrate	7.66×10^{3}
200 nm Ag + 60 min N_2	1.02×10^{5}
200 nm Ag + 45 min H_2	2.53×10^{3}
200 nm Ag + 30 min Ar	4.75×10^{3}
200 nm Ag + 15 min air + 15 min Ar	1.97×10^{4}
200 nm Ag + 400°C/15 min + 15 min air + 15 min Ar	2.35×10^{5}
200 nm Ag + 15 min O_2 + 20 min H_2	2.00×10^{4}

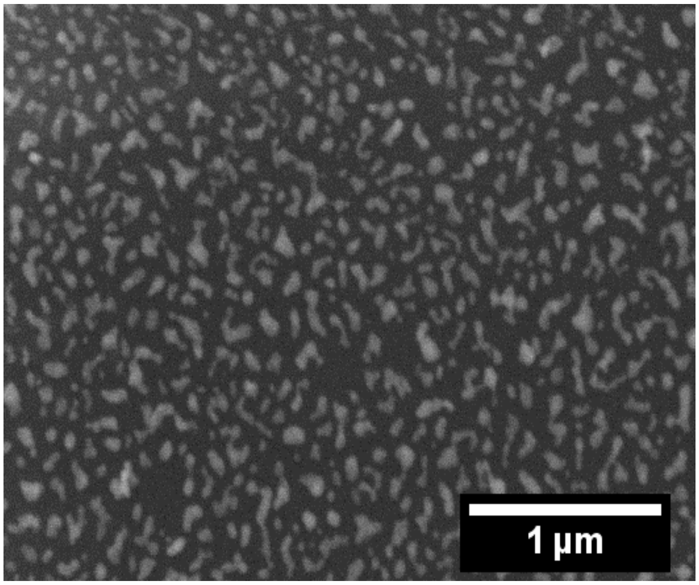

Figure S20: SEM image of 10 nm Ag treated with oxygen plasma (g12-p200) for 2 min followed by reduction with argon plasma (g16.7-p200) for 5 min.

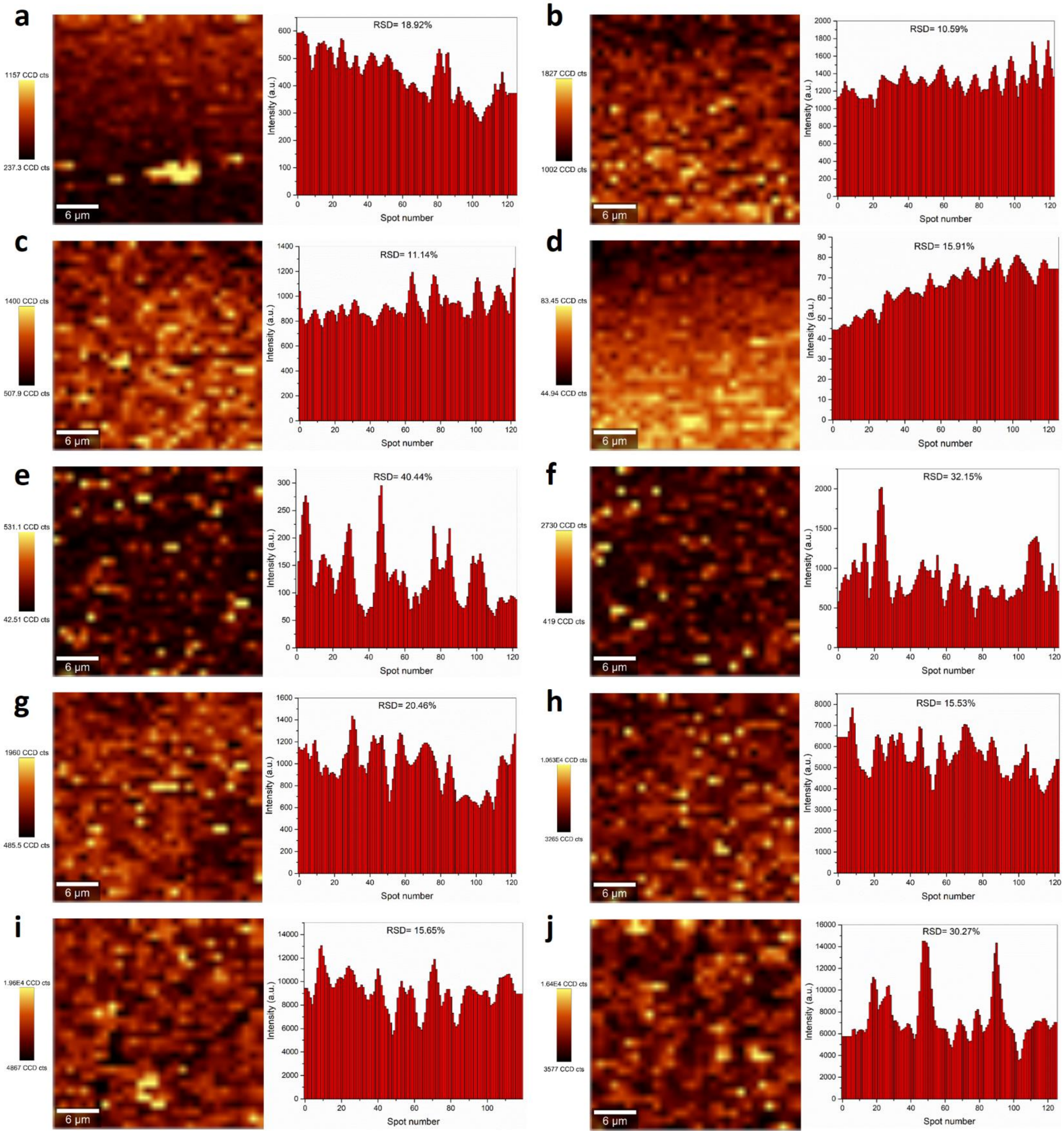

Figure S21: Raman mapping of the vibration at 1650 cm^{-1} and the corresponding intensities obtained from a section of the map of 10^{-6} M RhB on (a) 10 nm Ag, (b) 10 nm Ag + 2 min H_2 plasma, (c) 10 nm Ag + 10 min Ar, (d) 200 nm Ag, (e) 200 nm Ag + 30 min H_2, (f) 200 nm Ag + 30 min Ar, (g) 10 nm Ag + 2 min O_2 + 3 min H_2, (h) 10 nm Ag + 2 min H_2 + 5 min Ar, (i) 200 nm Ag + 15 min H_2 + 20 min O_2 and (j) 200 nm Ag + 15 min air + 5 min Ar.

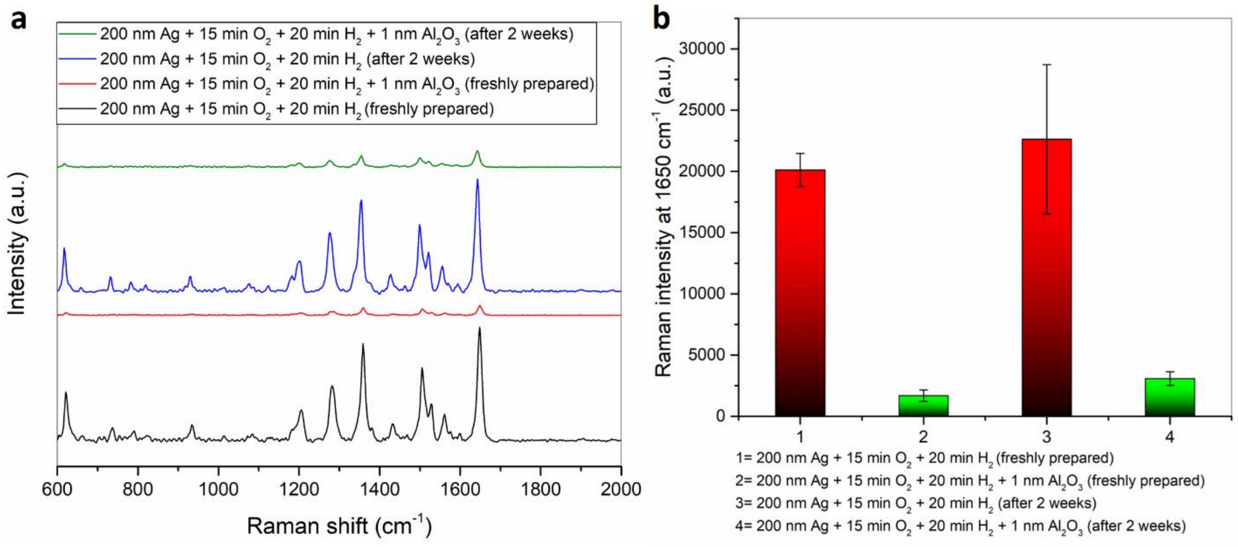

Figure S22: (a) Comparison of the SERS spectra of 10^{-6} M RhB on SERS substrates prepared through oxidation of 200 nm silver film with oxygen plasma (g12-p200) for 15 min followed by reduction with hydrogen plasma (g12-p200) for 20 min with and without 1 nm Al_2O_3 on top using 532 nm laser as excitation source. One set of substrates was directly used after preparation and the other set was used after 2 weeks from preparation. (b) Comparison of the average Raman intensities at 1650 cm^{-1} on the corresponding substrates.

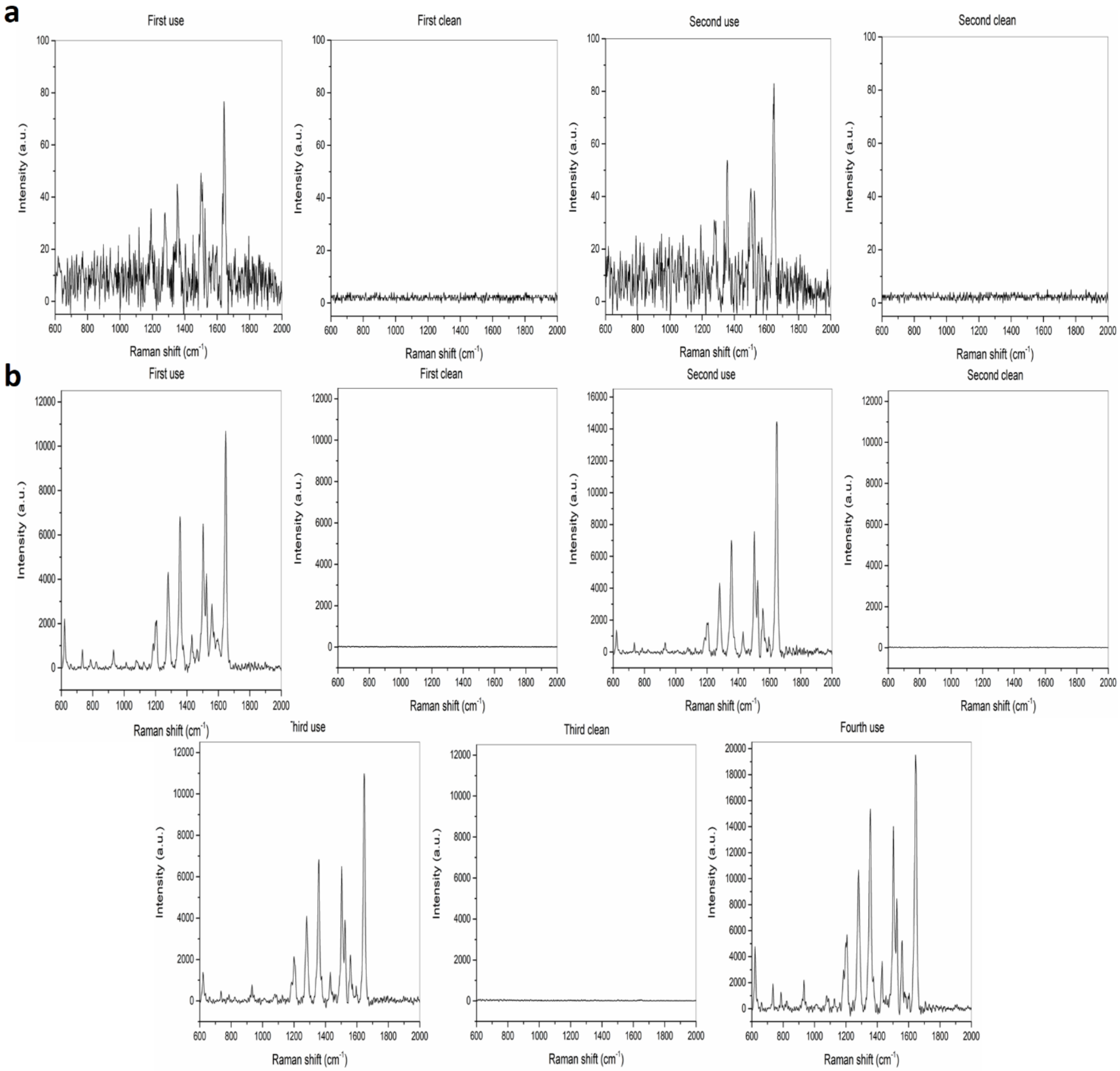

Figure S23: SERS spectra of 10^{-6} M RhB on (a) 200 nm silver film used as SERS substrate before and after cleaning with argon plasma (g16.7-p50) for 5 min for two cycles and (b) a six months old 200 nm Ag + 15 min O_2 + 20 min H_2 plasma (g12-p200) SERS substrate before and after cleaning with argon plasma (g16.7-p50) for 5 min for four cycles demonstrating the cleaning ability of argon plasma for SERS substrates.

References

1. El Mel, A.-A.; Stephant, N.; Hamon, J.; Thiry, D.; Chauvin, A.; Chettab, M.; Gautron, E.; Konstantinidis, S.; Granier, A.; Tessier, P.-Y. *Nanoscale* **2016,** *8*, 141–148. doi:10.1039/C5NR07145C
2. Waterhouse, G. I. N.; Bowmaker, G. A.; Metson, J. B. *Appl. Surf. Sci.* **2001,** *183,* 191–204. doi:10.1016/S0169-4332(01)00561-X
3. Zhou, X.; Zhou, F.; Liu, H.; Yang, L.; Liu, J. *Analyst* **2013,** *138,* 5832. doi:10.1039/c3an00914a
